# Toward implantable devices for angle-sensitive, lens-less, multifluorescent, single-photon lifetime imaging in the brain using Fabry–Perot and absorptive color filters

**DOI:** 10.1038/s41377-022-00708-9

**Published:** 2022-01-24

**Authors:** Adriaan J. Taal, Changhyuk Lee, Jaebin Choi, Björn Hellenkamp, Kenneth L. Shepard

**Affiliations:** 1 Columbia University - Department of Electrical Engineering, 500W. 120th St., Mudd 1310, New York, 10027 NY USA; 2 Korea Institute of Science and Technology - Brain Science Institute, 5 Hwarang-ro 14-gil, Seongbuk-gu, Seoul 02792 Republic of Korea

**Keywords:** Optoelectronic devices and components, Integrated optics, Nanocavities

## Abstract

Implantable image sensors have the potential to revolutionize neuroscience. Due to their small form factor requirements; however, conventional filters and optics cannot be implemented. These limitations obstruct high-resolution imaging of large neural densities. Recent advances in angle-sensitive image sensors and single-photon avalanche diodes have provided a path toward ultrathin lens-less fluorescence imaging, enabling plenoptic sensing by extending sensing capabilities to include photon arrival time and incident angle, thereby providing the opportunity for separability of fluorescence point sources within the context of light-field microscopy (LFM). However, the addition of spectral sensitivity to angle-sensitive LFM reduces imager resolution because each wavelength requires a separate pixel subset. Here, we present a 1024-pixel, 50  µm thick implantable shank-based neural imager with color-filter-grating-based angle-sensitive pixels. This angular-spectral sensitive front end combines a metal–insulator–metal (MIM) Fabry–Perot color filter and diffractive optics to produce the measurement of orthogonal light-field information from two distinct colors within a single photodetector. The result is the ability to add independent color sensing to LFM while doubling the effective pixel density. The implantable imager combines angular-spectral and temporal information to demix and localize multispectral fluorescent targets. In this initial prototype, this is demonstrated with 45 μm diameter fluorescently labeled beads in scattering medium. Fluorescent lifetime imaging is exploited to further aid source separation, in addition to detecting pH through lifetime changes in fluorescent dyes. While these initial fluorescent targets are considerably brighter than fluorescently labeled neurons, further improvements will allow the application of these techniques to in-vivo multifluorescent structural and functional neural imaging.

## Abstract (web summary)

An implantable imaging shank is designed and fabricated which enables multicolor lens-less light-field fluorescence imaging, in addition to measuring fluorescent lifetime through time-correlated single-photon counting.

## Graphical abstract

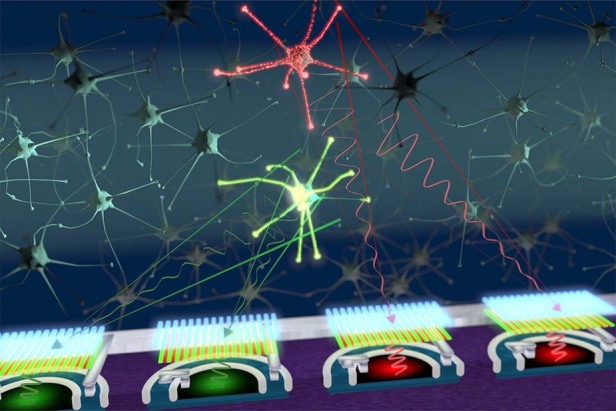

## Introduction

Optical imaging has revolutionized neuroscience by allowing recording of neural function in vivo. However, light scattering and absorption in tissue fundamentally limits the depth at which fluorescence microscopes can detect labeled neurons. To overcome this depth limitation, techniques to date have focused on microendoscopy, in which an optical fiber is implanted in targeted brain regions, sometimes with a miniature lens or prism to allow imaging at the fiber distal end^1,2^. These approaches, however, result in a significantly restricted field of view for a significant amount of tissue damage. At the same time, several realizations of head-mounted microscopes for 1p and 2p calcium imaging in mice have proven the feasibility of fluorescence microscopy with compact form factors^1,3–8^. To achieve imaging at depth, these instruments also require implantation of their requisite GRIN lenses (typically, with 0.3–2 mm diameter); this results in even greater displacement of brain tissue while still delivering only restricted fields of view. The limitations with existing state-of-the-art imaging approaches at depth have led to the search for alternatives in which the imager itself is given a shank form factor and directly inserted into the tissue^9,10^.

In this case, minimizing the thickness of the device is of prime importance to reduce displaced tissue volume. The lenses and filters associated with traditional microscopes are incompatible with this thin form factor. Instead, interest has turned to plenoptic imaging^11^, in which light conditioning is introduced before the photodetector to modulate its response based on incident angle, wavelength, arrival time, or polarization. Lens-less imaging^12,13^ is one form of light-field microscopy (LFM), which has recently benefited substantially from advances in plenoptic imaging capabilities^14^. When a diversity of pixels with maximally orthogonally modulating responses are combined computationally and the number of sources is constrained to be below the number of detectors^15,16^, compressive sensing algorithms allow for unambiguous reconstruction of the sources. Targeted labeling of functional neural reporters can provide the spatial-temporal source sparsity required to guarantee reconstruction. In general, the challenge for implantable imagers is to maximize signal conditioning to allow for application-specific image reconstruction, while aggressively minimizing the system footprint. While implantable lens-less methods lack the light gathering capabilities of lenses, they can image sources close to the device to compensate for diminished photon yield, not limited by the working distances associated with refractive optics.

One approach to modulate light to enable lens-less, volumetric, light-field imaging is with coded apertures^17,18^, either in phase or amplitude. These approaches allow for high-resolution imaging but still rely on placing these masks many wavelengths away from the photodetector. Pixel-to-pixel orthogonality is determined by the mask feature size and separation distance between the mask and the detector. The mask minimum feature size is limited by diffraction and thus imaging resolution will decrease when a thinner device is required.

A near-field diffraction approach based on the Talbot effect places diffraction gratings in front of the detectors^19,20^. Angle sensitivity is achieved with a pair of gratings: a diffractive grating and an analyzer grating. The top grating diffracts incident light into a Talbot diffraction pattern^21^, while the bottom analyzer grating selectively transmits or rejects the formed peak intensities, giving rise to angular modulation. In this case, distances between mask and detector are on the order of the wavelength, allowing lens-less imaging to be achieved with much thinner devices, opening up applications for flexible^22^, stackable^23^, or implantable imagers^24^. Amplitude-modulating gratings, which can be fabricated directly in the back-end metal of a conventional semiconductor fabrication process, produce this angular sensitivity but with significant optical loss. Phase gratings do not produce this signal loss but generally require an extra post-processing step^19^. These near-field diffraction approaches have an angular sensitivity that operates at a given wavelength with other wavelengths operating at degraded resolution^25^. Approaches to preserve resolution with shifted wavelengths such as odd-symmetry phase gratings^26^ show reduced angle-sensitive modulation generally.

In this work, we make these near-field conditioning masks angle-sensitive and multispectral, allowing multiple colors to be detected with a single photodetector within the context of angle-sensitive lens-less imaging. In this case, simultaneous spectral and spatial filter can be achieved. This is done by replacing the analyzer grating in the traditional angle-sensitive design with a dual-bandpass filter based on a metal–insulator–metal (MIM) Fabry–Perot structure^27–30^. We apply these masks to a complementary metal-oxide-semiconductor (CMOS) imager array, based on time-gated single-photon avalanche photodiode (SPAD) detectors^24^. The 1024-pixel imager consists of four 130 µm wide shanks (with 256 SPADs per shank), thinned to 50 µm thickness and packaged for neural implantation. We demonstrate how the combination of angular and spectral sensing allows for simultaneous source separation and localization of multispectral fluorescent sources. We demonstrate the localization of 45 μm diameter green fluorescent beads embedded in scattering medium. We also demonstrate lifetime imaging capabilities which further enhance source separability and provide the ability to estimate environmental pH using fluorescein lifetime. Imagers based on these techniques will eventually enable many interesting modalities for in vivo neural imaging.

## Results

### Diffraction grating constructed from metal–insulator–metal resonance filters

**Fig. 1:**
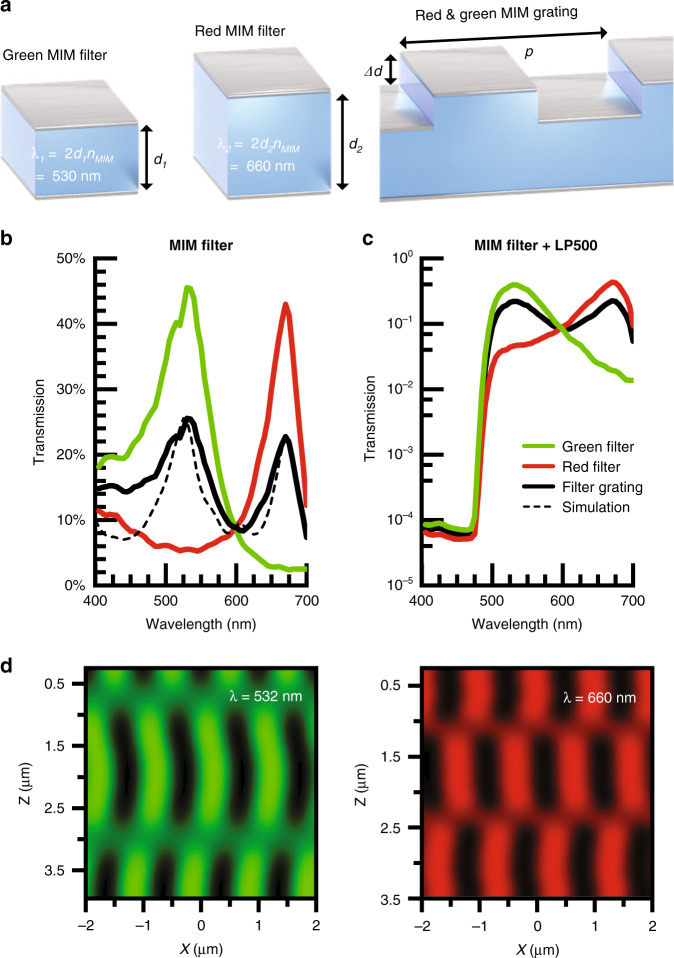
MIMAS filter design. **a** The Ag-HfO2-Ag MIM spectral filter selectively transmits a single resonant wavelength by tuning the oxide thickness. Interleaving both color filters with wavelength scale pitch constructs the grating structure. **b** Transmission spectrum of planar bandpass filters and interleaved grating filter over the visible spectrum for measured (solid) and FDTD simulated (dash) devices. The planar bandpass filters selectively transmit a single color, whereas the interleaved structure transmits a superposition of the two. **c** A 500 nm cut-on longpass filter dye is combined with the MIM filter, attenuating wavelengths under 480 nm by four orders of magnitude. **d** Verification of non-overlapping microscale Talbot self-image using near-field scanning optical microscopy (NSOM) in transmission mode, at wavelengths of 532 and 660 nm.

Key to this design is the MIM analyzer grating. As opposed to plasmonic hole and nanodisk array filters^31–34^, these MIM structures do not depend on spatial periodicity and thus can be laterally patterned on the wavelength scale. We begin with two MIM filters, green and red bandpass filters designed with HfO_2_ dielectric thicknesses of *d*_1_ = 70 nm and *d*_2_ = 100 nm, respectively, sandwiched between two 30 nm thick silver layers (Fig. 1a). We define the depth difference, *Δd* = *d*_2_−*d*_1_. The optical cavity selectively transmits light with a wavelength twice the optical path length, which is determined by the product of the insulator refractive index and thickness. In this case, we create a dual-bandpass filter that also functions as a diffraction grating by turning the top mirror of the constituent filter design into a grating (chosen to have a pitch, *p*, of 1 µm pitch) and interleaving these gratings for the red and green filters.

Figure 1b shows the measured spectral transmission efficiency for each structure in Fig. 1a under normal incidence (with the incident angle *θ* = 0°). As expected, the interleaved grating transmits a superposition of the individual planar MIM bandpass filters. Finite-difference time-domain (FDTD) simulations (ANSYS Ltd, Waterloo, Canada) show similar performance (Fig. 1b) for these structures (Supplementary section S1) with peak transmissivity in excess of 25% at thicknesses of 150 nm. More importantly, the red filter rejects green light by a factor of 10, and vice versa, minimizing cross-talk to allow for robust separation of these color channels. Fabrication nonidealities lead to lower rejection in the blue regime experimentally for the interleaved filter. This can be compensated, in part, by the introduction of a 3.5 µm thick layer of SU-8 epoxy containing an absorptive dye which provides a 500 nm cut-on longpass filter characteristic. As shown in Fig. 1c, wavelengths under 475 nm are attenuated up to four orders of magnitude. The MIM filter additionally blocks autofluorescence generated by the absorptive dye. This SU-8 layer also acts as the spacer between the diffractive and analyzer gratings in the full angle-sensitive implementation.

The full width at half maximum (FWHM) determines how closely spaced two resonant colors can be designed (Supplementary section S2) while maintaining two well-defined bandpass characteristics. The filter *Q*-factor (defined as the ratio of peak-transmission wavelength, λ_0_, to the FWHM) is determined by metal reflectivity (*R*) as

1 $$Q = \frac{{\lambda _0}}{{{\rm{FWHM}}}} = \frac{{\pi \sqrt R }}{{1 - R}}$$

For the green and red bandpass filters of Fig. 1b, *Q*-factors of 8.7 and 10.4, respectively, are achieved, consistent with the high reflectivity (*R* ≈ 0.7) of the silver films. These *Q*-factors match the emission spectrum of popular fluorescent proteins, such as EGFP, Alexa 488, and FITC. HfO_2_ (*n*_MIM_ = 2.1) was chosen over SiO_2_ as the insulator material to decrease the sensitivity of the transmission spectrum to *θ* and *p* variations (Supplementary section S3).

The near-field diffraction produced by these interleaved filters can be directly measured through the use of near-field scanning optical microscopy (NSOM)^35^. In these measurements, local electric fields are transmitted through a transparent-tip cantilever and coupled into a fiber through a ×100 objective. The light intensity is recorded using a photomultiplier tube. NSOM measurements at 532 nm and 660 nm produce non-overlapping Talbot diffraction effects in a 4 × 4 μm area above the gratings (Fig. 1d). We verify the measurements with FDTD simulations (Supplementary section S4).

### Angle-spectral-sensitive pixel front end on CMOS

**Fig. 2:**
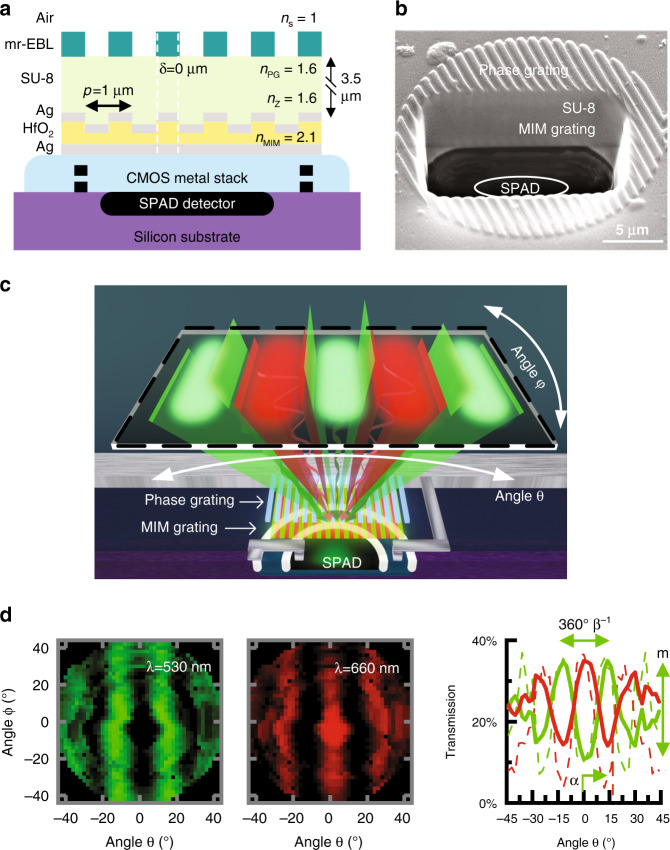
MIMAS filter integrated on CMOS. (**a**) Fabrication of MIMAS on CMOS (**b**) FIB-SEM sideview showing front end fabrication, with annotations for SPAD, electrical contacts and CMOS metal stack. **c** The dual grating structure selectively transmits light according to incident angle, illustrating the point spread function from a single detector for either wavelength. **d** Two-dimensional angular response for a single device for both resonant wavelengths. A plot at *φ* = 0° compares measurement (solid) to simulation (dash). Resulting parameters of interest are the angular period 360° *β*^−1^, peak transmissivity offset from zero *α*, and modulation index *m.*

To create these MIM angle-sensitive (MIMAS) conditioning masks, the MIM filter grating structure is used as the analyzer grating, while a phase, rather than amplitude, diffraction grating is used, doubling transmission efficiency and minimizing internal reflections to produce sharper angular modulation. We fabricate such grating structures on top of a CMOS SPAD photodetector^24^ as shown in Fig. 2a.

To fabricate this, we sputter through a shadow mask a 30 nm thick Ag layer and a 100 nm thick HfO_2_ insulator. Subsequently, we etch the HfO_2_ after defining the pixel-specific mask in PMMA through electron-beam lithography. Then, we sputter deposit 25 nm thick Ag capped by 30 nm of HfO_2_ to prevent oxidation of the silver. The LP500 filter dye (Adam Gates Company, Hillsborough Township, NJ, USA) is dissolved in 3.5 µm thick SU-8, separating the MIM grating from the phase Talbot grating directly patterned in 220 nm thick mr-EBL negative-tone electron beam resist^36^. This resist provides the high refractive index (*n*_PG_ ≥ 1.6) required for a suitable refractive index contrast with the source medium (*n*_PG_*-n*_s_), either air or aqueous media.

Figure 2b shows the scanning-electron microscope (SEM) image of a postprocessed SPAD taken after focused-ion-beam-milling cross-sectioning (FIB-SEM). The SPAD, MIM grating, SU-8 separation layer, and phase grating are annotated. Despite the thickness of the silver, its high reflectivity makes the MIM grating structure visible. The refractive indices of the surrounding medium, phase grating, separation layer, and MIM dielectric are given by *n*_s_, *n*_PG_, *n*_Z_, and *n*_MIM_, respectively. The epoxies SU-8 and mr-EBL have been studied for cytotoxicity, with results varying from fully biocompatible to slightly cytotoxic^37,38^. While the SU-8 layer can be encapsulated, additional encapsulation is difficult because of the need to have abrupt index features in the mr-EBL layer.

In conventional lens-based imaging, each pixel is mapped to a unique point on the object focal plane. In contrast, a lens-less imager that uses angle-sensitive pixels compressively samples the scene using co-sinusoidal basis functions (Fig. 2c), similar to a discrete cosine transform^39^. The function describing this pixel-level modulated sampling is defined as *T*(*θ,λ*) where *θ* denotes the elevation angle pivoting around the *x* axis. We can parameterize *T*(*θ,λ*) with angular frequency *β*, peak offset from zero *α*, modulation index *m*, and grating rotation χ as

2 $$T\left( {\theta ,\lambda } \right) = \frac{1}{2} + \frac{m}{2}\cos \left[ {\beta \left( \lambda \right) \cdot \theta \left( {\upchi} \right) + \alpha \left( \lambda \right)} \right]$$

after we convert the volume of interest, relative to the pixel location, from Cartesian to spherical coordinates (Supplementary section S5).

The pixel response is fully characterized by a far-field angular measurement. The device is mounted on two rotational stages to measure 2D angular modulation. Figure 2d shows measured angular (*θ* and *φ*) dependency and detailed simulated and measured *θ*-angle-dependent transmission efficiency (*χ* = 0) at *φ* = 0°. The measurements are normalized against a SPAD imager without fabricated gratings, showing a 32% peak-transmission efficiency, compared to the 35% predicted in simulation. A 25% average transmission over all angles is observed. In these measurements, we observe the generation of orthogonal angular-sensitivity functions at each color. The green (530 nm) and red (660 nm) responses are phase shifted with respect to each other by *α*=180°, a direct result of the interleaved color filter gratings. The experimentally found values for *β* (11 and 12.5 for green and red, respectively) compare well to simulation. Incident light is correctly modulated up to ±45°. At higher incident angles, transmissivity is decreased due to occlusion from the CMOS metal stack. In aqueous environments, the angular frequency *β* increases^19^ by a factor *n*_s_·*n*_air_^*−*1^ = 1.33. The modulation index, *m*, determines the sensitivity of the response to changes in the angle of incidence. We achieve *m*~0.65, close to those achieved in simulation (*m*~0.8). These results show we can achieve a well-defined, pixel-level angular-spectral modulation of incident light with these masks. The pixel-to-pixel standard deviation in *m* for a given angular-sensitive geometry is typically less than 0.06 (also Supplementary section S5). As the angle of maximum intensity recorded from each pixel does not see significant pixel-to-pixel variability and this has a more dominant influence on reconstruction quality, we do not see significant effects on image reconstruction associated with using the average transmission for each incident angle for parameterization.

### Array-wise response of imager array

**Fig. 3:**
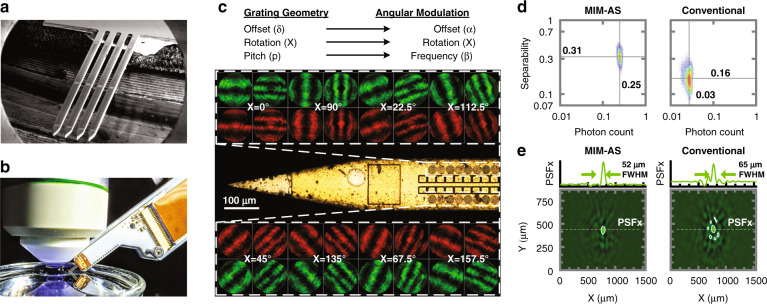
MIMAS-CMOS implantable shanks. **a** The implantable imager consists of four shanks, each employing 2 × 128 SPAD pixels. **b** The chip is packaged and mounted on a micromanipulator for implantation. **c** Photomicrograph showing the 16 front end geometries distributed over the imager. Rotations (*χ*) are swept along the *x*-direction, grating pitch (*p*) and offsets (*δ*) placed adjacent. **d** The linear separability and relative photon count (compared with a SPAD with no mask) for all voxels in a 1000 × 1000 × 150 µm subset of the field of view. The MIMAS (current work) is compared with a conventional metal-based angle-sensitive design where each pixel carries separate red and green filters for each pixel alternatively. **e** PSF in the *XY* plane at a height of 150 µm above the pixel ensemble with detail along *y* = 400 µm. A conventional angle-sensitive array without dual-bandpass filter shows distorted PSF, as its effective pixel density is halved.

We fabricate these MIMAS frontends on an implantable CMOS imager, designed in a TSMC 130 nm high-voltage technology, which consists of four shanks (Fig. 3a). Each shank contains 2 × 128 SPAD pixels, with 24.5 µm and 92 µm pixel pitch in the *x* and *y* directions, respectively. The pixel pitch is chosen to support SPAD quenching and detection circuit, five-bit in-pixel memory, and global shuttering and readout. The SPAD geometries are chamfered squares with 14 µm vertices for a fill factor of 8%. The 700 µm long tip ensures a sharp insertion profile but does not carry detectors. The shank imager is wirebonded onto a circuit board, ready for neural implantation (Fig. 3b).

Each front end’s angular frequency, phase, and rotation correspond to a unique point in Fourier space. For the imager to sample the scene in the complete Fourier space, a diversity of pixels is required which orthogonally sample angular frequency (*β*), phase (*α*), and rotation (*χ*). While high angular frequencies *β*, implemented by small grating pitch, provide high spatial resolution, low angular frequencies are needed to complete the sampling spectrum^40^ and result from larger grating pitches. Simulated and fabricated values abide to a previously derived analytical model for β^19^. The implementation of grating rotations *χ* allows one to resolve rotated features (*θ*(*χ*)). Changing the grating offset *δ* allows the sampling of angular frequency with different phases (*α*). We design a repeating ensemble of maximally orthogonal front-end geometries, repeating every sixteen pixels along the array. This repetition ensures that a source located 150 μm above the shank is in the field-of-view (FoV) of each geometry.

Figure 3c shows the tip of the shank with the pixel array starting 600 µm from the tip, to ensure smooth insertion. The 16 different grating structures are created from two pitch sizes, two grating offsets, and eight grating rotations as structural design parameters. The 2D angular response for each pixel front end is shown next to the pixel for both red and green wavelength. A large diversity of rotations is found to be more beneficial in achieving orthogonality than pitch and offset variation (Supplementary section S6).

In this form factor, each of four shanks displaces a tissue volume of 120 × 4000 × 50 µm while yielding an imaging FoV of 200 × 4000 × 150 µm. In this shank geometry, the FoV and volume scale equally with an increasing number of pixels. Gradient refractive index (GRIN) lenses^41,42^ and implantable multimode fibers^43^ allow imaging at depth with external optics, imagers, and light sources. However, they image only from the end face, leading to a displaced volume that scales only linearly with both FoV and desired depth. Furthermore, because using these lenses still requires bulky external objectives and image sensor systems, fully implantable versions of these systems remain elusive. For the current shanks, the FoV-to-volume ratio is approximately five, two orders of magnitude better than what is achievable with implantable GRIN lenses. This will only improve with further advances in pixel sensitivity and with shank width and thickness optimizations.

### Multispectral sparse reconstruction formulation

Before imaging, we establish the computational framework for image reconstruction, making use of compressive sensing techniques. In this multispectral, lens-less imaging, the imaging volume is compressively sampled through a spatial sensing matrix *A*. Each element *A*_*ij*_ represents the response from voxel *j* to pixel *i*. As such, the rows *A*_*i*_ correspond to the co-sinusoidal modulation of each pixel and are constructed from the angular modulation function *T*_*i*_(*θ,ϕ,λ*). The response is converted from spherical to Cartesian coordinates and translated to the pixel’s position on the imager to allow inverse imaging in an arbitrary volume voxel grid. The sensing matrix *A* compresses the image *x* onto the recorded data *y* for each color (*A*_*g*_, *A*_*r*_). By solving for the image, the solution explicitly contains a source color classification (*x*_*g*_, *x*_*r*_):

3 $$Y\left( t \right) = Ax(t) + {\it{\epsilon }} = \left[ {\begin{array}{*{20}{c}} {A_g} & {A_r} \end{array}} \right]\left[ {\begin{array}{*{20}{c}} {x_g(t)} \\ {x_r(t)} \end{array}} \right] + {\it{\epsilon }}$$

where *ϵ* represents photon and dark-count shot noise, ignoring spectral cross-talk. While the data from each pixel *y*_*i*_ are determined from multiple sources *x*_*j*_, sparse reconstruction theory states that a given number of recording sites allows localization of a smaller or equal number of sources, even though the number of voxels, and thus the dimensionality of the problem, may be much higher. For the MIMAS imager, the out-of-phase angular transmission spectrum forces orthogonality of the *A*_*g*_ and *A*_*r*_ submatrices, supplying the information required to correctly identify point sources of multiple colors.

The quality of localization of each voxel within the field of view can be quantified using two metrics, the photon count and the linear separability. The photon count from a voxel (relative to a SPAD imager in the absence of the MIMAS mask) determines the achievable signal-to-noise ratio (SNR). The linear separability for each voxel *j* is quantified by the cosine of the angle between the *j*th column of the sensing matrix *A*, *A*_*j*_, and the hyperplane spanned by all other vectors in the matrix. Separability is expected to decrease with voxel density. We calculate both metrics for each voxel within a subset of the field of view, generated from a 1000 × 1000 × 150 µm volume at 15 µm rectangular grid.

Figure 3d compares linear separability and photon count between the current MIMAS design and a metal-grating-based angle-sensitive design. The dual-bandpass solution provides double the effective pixel density for each color channel as compared to a metal-based angle-sensitive array with separate red and green filters for each pixel. For MIMAS, the spectral sensitivity and added orthogonality between both color channels provide a 95% higher separability value. A 25% transmission efficiency over all angles, compared with 3% previously reported for metal gratings^9^, improves signal photon count by over 8×.

For the metal-grating-based angle-sensitive implementation, there is already limited ability to perform spectral decomposition. However, the green (*A*_*g*_) and the red (*A*_*r*_) sensing submatrices are highly correlated (Supplementary section S7). This provides an insufficient amount of color sensitivity to cope with the doubled dimensionality of the multispectral inverse imaging problem.

The minimum distance between two neighboring point sources, however, as recovered by a sparsity-regularized least-squares solver, is limited by the dimension of the point spread function (PSF). We calculate the PSF, as seen by the pixel ensemble, around a point of interest *x* by applying the projection matrix *P*_*A*_:

4 $${\rm{PSF}}_g = P_{A_g} \cdot {{{\mathrm{x}}}} = A_g^{\rm{T}}\left( {A_gA_g^{\rm{T}}} \right)^{ - 1}A_g \cdot {{{\mathrm{x}}}}$$

Figure 3e shows how a simulated point source, centered above the pixel ensemble at a height of 150 µm, resolves to a point spread function for the green color. The structure of the PSF is a combination of co-sinusoids as a result of the angular sensitivity, shown in detail along the *x* dimension. Consequently, the PSF confinement in the *xy*-plane depends strongly on the *z* location. Objects closer to the imager will, therefore, enjoy higher resolution^9^. While the PSF has a FWHM of 52 µm, its central peak at the source location is unbiased, enabling sparse source localization. Figure 3e also shows the severely aliased spatial confinement of the PSF of an array where each pixel is alternatively red and green filtered.

### Fluorophore demixing and imaging

**Fig. 4:**
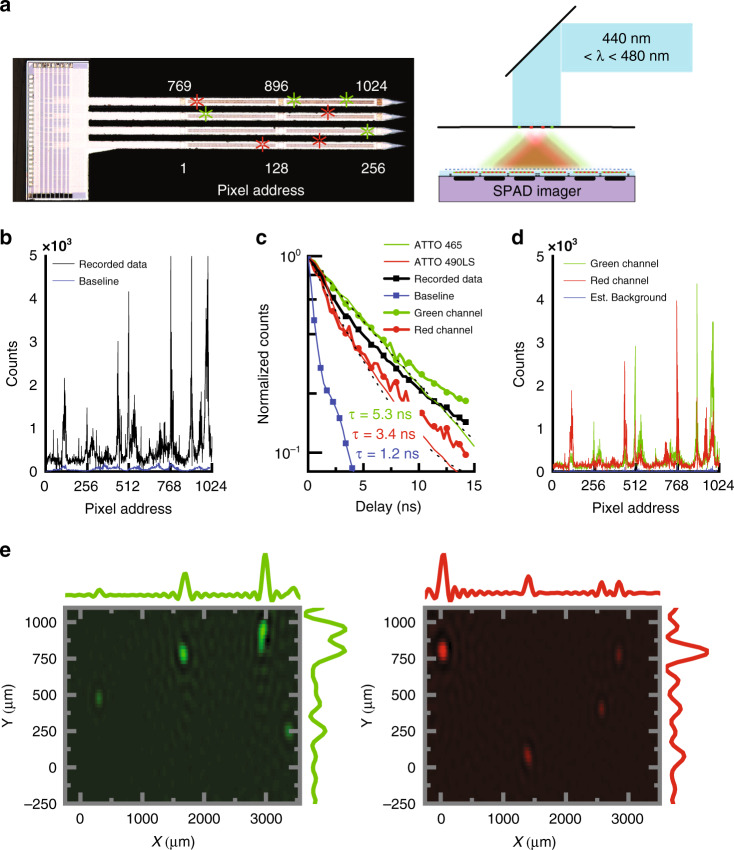
Multi-wavelength lens-less imaging using angle-sensitive pixels. **a** Photomicrograph of the fluorescent pinhole array overlaid on the chip. **b** 30 µMol l^−1^ ATTO 465 and ATTO 490LS fluorescent dye is dispersed in 1.5% agar simulating scattering tissue. The agar is overlaid on a pinhole array and illuminated from the back. **b** Recorded data from MIMAS imager chip. **c** Calibration of recorded lifetimes for 30 µMol l^−1^ green, red and undyed agar. Time series for the mean recorded data, separated in green and red color channels through blind source separation. **d** Data separated into color channels after blind source separation. **e** Inverse image for both wavelength channels with detail plots representing the summation in the *X* and *Y* dimensions.

In order to validate the MIMAS imager in a context relevant to neural imaging, we demonstrate simultaneous detection of multispectral fluorescent sources. We do this first by placing a pinhole array carrying agar stained with a fluorescent dye 400 µm above the imager (Fig. 4a). The pinholes are alternatively stained with ATTO 465 (maximum emission 515 nm, lifetime 5.0 ns) and ATTO 490LS (maximum emission 660 nm, lifetime 2.6 ns) as proxies for fluorophores such as GFP and RFP.

An NKT EXU-6PP mode-locked supercontinuum laser carries 1.5 mW of average power at 19.5 MHz repetition rate, and illuminates the field of view through the pinhole array. The pinhole array location and rotation are oriented such that the individual pinholes are within the field of view of a maximum number of pixels. The output from the SuperK Varia acousto-optic transmission filter was tuned to 460 nm with 40 nm bandwidth to approximately equally excite ATTO 465 and 490LS, accounting for respective excitation spectra and quantum yields. Even though the two dyes exhibit a 5% emission overlap in the yellow regime, the MIM filter attenuates cross-talk to less than 1% of total signal power. The majority of blue excitation light is not converted to fluorescent signal and is thus projected on the imager as background. In order to maximize signal yield, we increase the laser power to the point at which we begin to observe pile-up in the SPADs, which typically occurs when a photon arrival event occurs in more than 3% of all SPAD frames^9^.

The imager records the data *Y* (Fig. 4b) with a frame rate of 10 Hz. We select this frame rate as it is the minimum required for functional neural imaging. Data from “hot pixels” (more than five times median dark counts) are discarded. Regions in the data with both high intensity and strong modulation are indicative of a source. The combination of the MIM and absorption filter rejects blue excitation light with OD 3, while the angular sensitivity rejects the spatially uniform background excitation light with an additional OD 0.8. In contrast, fluorescent light, entering at an angle of maximum transmission, is transmitted through the MIMAS with over 30% efficiency. A baseline recording in absence of fluorescent sources is plotted for comparison.

By time-gating the SPAD detectors (enabling them after the laser has been shut off) with a shifting 20% duty-cycle enable signal, we can measure fluorescence lifetime^9,44^. Figure 4c shows calibration lifetime measurements for uniformly stained 10 µMol l^−1^ ATTO 465 and 490LS fluorescent dye in 1.5% w/v agar in phosphate buffered saline (PBS, pH 7.4), representative of neural tissue densely labeled with EGFP^45^. The estimated lifetimes for the green (5.3 ns) and red (3.4 ns) dyes are a convolution of the dye lifetimes (5.0 ns, 2.6 ns) and the lifetime of the SPAD instrument response function (IRF) (1.2 ns).

The lifetime characteristics of the fluorophore aid in robust localization of the fluorescent sources even in the presence of insufficient excitation light rejection. Separation is further aided by the presence of more than one spectrally distinct fluorophore of different lifetimes. Using orthogonal non-negative matrix factorization (O-NNMF)^46^, we incorporate the calibrated lifetimes as a priori information to correctly separate the recorded data *Y*(*t*) into green and red channels in *S*(*t*) (Fig. 4c) and their associated data in *H*_*g*_ and *H*_*r*_ (Fig. 4d):

5 $$Y\left( t \right)\mathop { \Longrightarrow }\limits^{{\rm{O}}{\mbox{-}}{\rm{NNMF}}} H \cdot S\left( t \right) + {\it{\epsilon }} = \left[ {\begin{array}{*{20}{c}} {H_g} & {H_r} \end{array}} \right]\left[ {\begin{array}{*{20}{c}} {S_g(t)} \\ {S_r(t)} \end{array}} \right] + {\it{\epsilon }}$$

Even though neither *S*(*t*) nor *H* supply any spatial information, the source separation acts as a matched filter in the temporal domain, removing background excitation photon counts in ε and enhancing SNR in *H*. This blind source separation (BSS) is only possible as *H*_*g*_ and *H*_*r*_ span orthogonal subspaces because of the properties of the MIMAS conditioning masks. The separated red and green channel have a total correlation of 0.46 over the entire data sequence. This correlation is directly related to the correlation between the two lifetimes. The estimated background is small and has a fast lifetime decay (1.2 ns), equal to the SPAD IRF.

We make a best guess for *L*, the total number of fluorophore locations, by looking at the singular value decomposition of *Y*. We localize the fluorophore locations for each color channel separately using the sparsity-constrained in-crowd algorithm^47^. Afterward, an inverse image is formed by applying the projection matrix to the *L* most likely locations. Figure 4e shows the inverse image for *L* = 8. All pinhole locations are found and show local maxima at the true locations. The circular aliasing artifacts in the image are a direct result of the combination of rotated angle-sensitive pixel responses, as seen in the structure of the PSF. The solution is extremely robust against variation in input parameter L (Supplementary section S8).

### Fluorescent beads in scattering medium

**Fig. 5:**
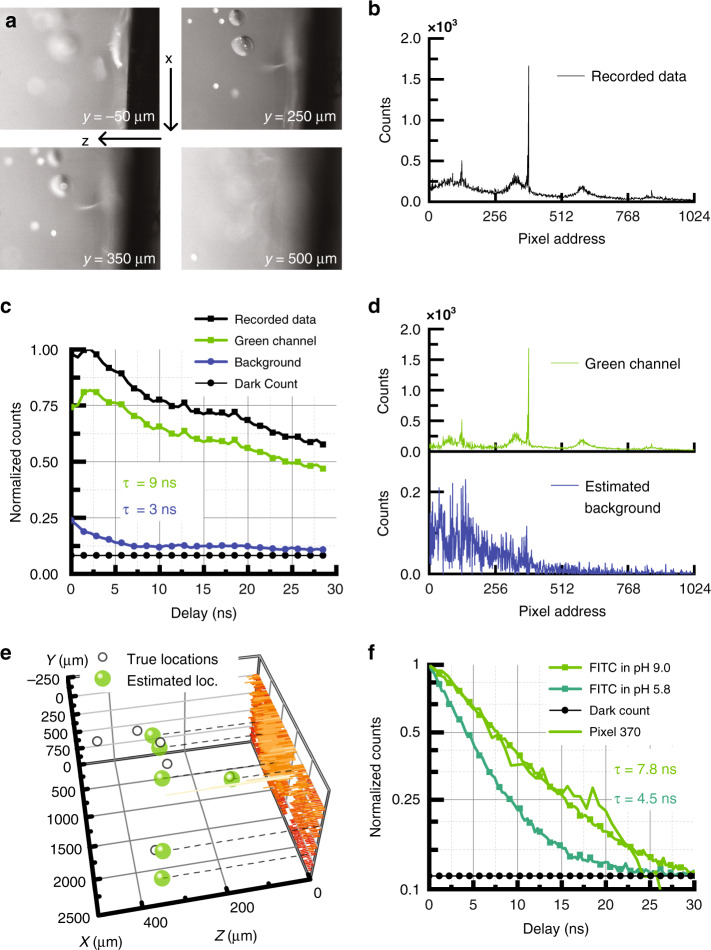
Localization in scattering medium. **a** Photomicrograph of 45 µm polymer beads containing Fluoresbrite YG, dispersed in 1.5% agar simulating scattering tissue. The probe is inserted at *z* = 0 orthogonal to the direction of excitation light. **b** Recorded data from MIMAS imager chip at 0 ns delay. **c** Time series for mean recorded data, estimated fluorescent signal and excitation background through blind source separation. **d** Estimated scattered illumination background and fluorescent data after blind source separation. **e** Volumetric localization through an *L* = 6 sparse representation, compared with fluorescent bead ground truth. **f** Lifetime calibration for FITC as a function of environment pH. The lifetime fingerprint of pixel 370 reveals the Fluoresbrite is encapsulated in an environment of pH = 9.

We next use a phantom model to verify that the MIMAS CMOS imager is capable of detecting localized fluorescent sources in scattering media. We insert the shank into 1.5% w/v agarose (Fig. 5a). The imager is positioned orthogonal to the direction of illumination (at *z* = 0 in Fig. 5a) to minimize excitation laser light directly entering the SPADs. Fluoresbrite YG beads with 45 µm diameter are randomly dispersed inside the agar. These beads provide the highest brightness while constraining dimensions within the resolution limit of the imager.

The laser illuminates the field of view through a ×20 water immersion objective. The brightfield image at four different depths reveals six beads inside the field of view of the shanks. The data, recorded at a frame rate of 10 Hz, is plotted in Fig. 5b. As the ×20 objective does not cover the entire field of view and agar scatters photons anisotropically, the illumination is not uniform. This will be characteristic of systems employing any kind of spatially localized illumination^10^. The emitter matrix, *E*, describes the illumination photon flux for each voxel and is incorporated in the linear system of equations:

6 $$Y\left( t \right) = A_gx(t)E + {\it{\epsilon }}$$

A simple model is computed using ValoMC^48^ based on the Henyey-Greenstein phase function for anisotropic scattering. We use a scattering length of 2.86 mm^−1^, refractive index of 1.34, and anisotropy parameter of 0.9^49,50^, closely resembling neural tissue^51,52^. Absorption inside the agar is considered negligible. We model a conical source with a 450 nm wavelength and 1000 µm FWHM, entering the agar from water with index 1.33.

**Fig. 6:**
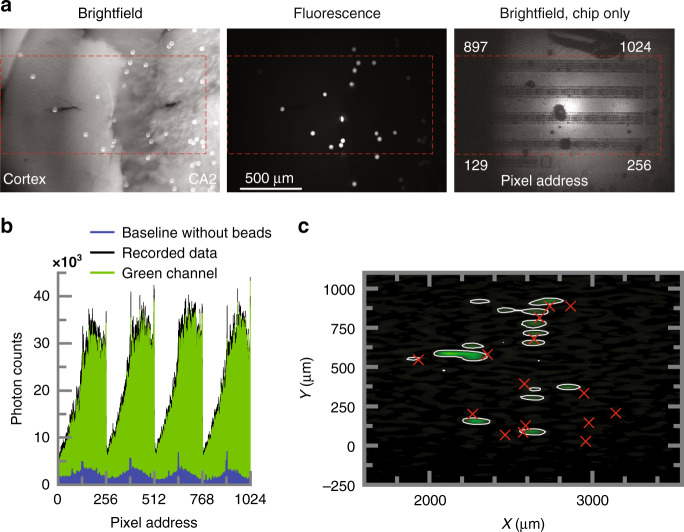
Ex-vivo recording. **a** Brightfield image of the whole brain slice laminated over the chip. The fluorescence image reveals the beads distributed through the tissue. Brightfield image of the location over the chip. **b** Recorded data, plotted alongside the background recorded for illumination of brain slice in the absence of beads. **c** Inverse image of the data recorded, compared to the true locations denoted by red crosses. The contour is drawn around all values corresponding to 10% of the maximum value of the image.

The source-separation technique for background estimation and removal is repeated. The long fluorescence decay is used to separate the data into fluorescence (9 ns) and background (3 ns) lifetimes (Fig. 6c). The average lifetime of the estimated background is longer than calibrated, as the inherently imperfect separation algorithm attributes some fluorescent photon counts to the background. The estimated fluorescent and background contributions are plotted in Fig. 6d. We localize the fluorescent sources using the sparsity-constrained least-squares solver. Figure 5e shows the localized fluorescent targets compared to ground-truth locations. The mean absolute Euclidian error averaged across the five identified sources is 99 µm, less than twice the FWHM. A sixth location is too far away to be found.

The effectiveness of temporal fluorescence separation is limited by the signal-to-noise ratio (SNR), given by

7 $${\rm{SNR}} = \frac{{N_{{\rm{fluo}}}}}{{\sqrt {N_{{\rm{fluo}}} + N_{{\rm{BG}}} + N_{{\rm{DCR}}}} }}$$

where *N*_fluo_, *N*_BG_ and *N*_DCR_ are the fluorescence, excitation background and dark noise photon counts under Poisson photon arrival statistics, respectively^53,54^. At best, we can subtract a per-pixel estimate for the background but not its induced shot noise. The pixel with peak signal (pixel 370) registers a maximum SNR of 32 dB. Over the whole imager the average SNR is 17 dB. The spatial reconstruction accuracy of a single fluorescent source is limited by the SNR, the MIM filter FWHM, and fluorophore emission spectrum (Supplementary section S9).

Finally, we demonstrate how the chip can exploit lifetime imaging to optically measure pH levels^55^. Functional groups of fluorescein are modulated by pH, modulating the fluorescent lifetime^56,57^. Inside neural tissue, local variations in pH could indicate glial cell misfunction^58^, activation of pain signaling^59^, or can even be used to directly image synaptic activity^60^. State-of-the-art implantable pH sensors based on electrodes offer low electrode count in a narrow field of view^61^, while sensors based on diffusion dynamics suffer from unpractically long acquisition times^62,63^. The optical paradigm could offer fast framerates and a large field of view.

As a calibration, we dissolve 10 µMol l^−1^ in 1.5% w/v agar using PBS with pH levels of 5.8 and 9.0. Figure 6e shows how the FITC lifetime increases strongly with environment pH. The lifetime as seen by the imager ranges from 4.5 ns (pH = 5.8) to 7.8 ns (pH = 9). We compare these calibrations with the lifetime registered by pixel 370 in the Fluoresbrite experiment, which correlates strongest with the calibrated lifetime for pH = 9. By these means, the imager can simultaneously image and infer environmental pH of a fluorescent target.

### Ex-vivo fluorescent imaging

We image fluorescent beads ex-vivo through a whole-brain slice to verify operation inside strongly scattering tissue. The 150 μm thick coronal mouse brain slices are contained within a 3D printed mold and perfused with artificial cortical spinal fluid (ACSF). We position 45 µm FITC beads on the side of the tissue opposite the shank (Fig. 6a) to emulate clusters of densely intracellularly dyed neurons^64^. The brightfield image reveals 15 beads within the red annotated field of view. The tissue structures are also clearly visible. For excitation, a supercontinuum laser is tuned to a spectrum of 480–490 nm and is projected onto the slice through a Nikon 4×0.15NA objective. As can be seen in the fluorescent image, the illumination profile is constant over an 800 μm diameter area. Uniform illumination is key when imaging over very large fields of view.

We compare photon counts from slices both with and without beads present (Fig. 6b), and find that the c background (primarily resulting from unfiltered excitation photon scattered into the detectors) is about 10× dimmer than the fluorescent signal from the beads. We estimate the green fluorescent signal (“green channel”) by subtracting the background from the raw data for a maximum estimated SNR of 22 dB.

The resulting image of 15 source locations is shown in Fig. 6c. We find 10 sources with an average error of 200 μm, which corresponds to less than 2× the measured PSF FWHM for a single 45 μm bead in scattering tissue (Supplementary section S10). The other five beads are found as false positives in larger clusters next to correct locations. The imager also struggles to correctly identify beads illuminated less brightly by the laser. It is important to note that these 45-μm beads used here have a fluorescence that is approximately 100× brighter than what would be expected from the soma of a GCAMP6f transgenic neuron, for example. Further improvements in background rejection (approximately an additional OD2) will be necessary to observe fluorescence of the intensity required for GCAMP6f imaging.

## Discussion

In this work, we presented an implantable imager, based on an optical sensor front end that combines optical bandpass filters with angular selectivity for compressive sensing of two colors. The MIM grating, fabricated by interleaving two MIM bandpass filters, creates a superposition of two independent Talbot diffraction patterns. The MIMAS front end, fabricated by superimposing a phase grating above the MIM grating, provides well-defined, orthogonal angular modulation for two wavelengths.

The MIMAS conditioning mask increases multicolor sparse localization over conventional angle-sensitive designs employing metals for both diffractive and analyzer grating in three important ways. First, the interleaved color filter reduces color channel cross-talk within the same photodetector by a factor of 10, improving location and color classification of fluorescence point sources. Secondly, because of the use of phase diffractive grating, the color filter phase grating design is 15% efficient over all angles, compared to 3% using metal gratings. Third, the orthogonality between the two-color channel sensing submatrices enables blind source separation of fluorescent lifetimes, greatly enhancing removal of background fluorescence which is characterized by a different lifetime.

By augmenting a CMOS SPAD array with a diversity of MIMAS geometries, the correct localization and labeling of green and red fluorescent dyes were demonstrated. The MIMAS SPAD array was implanted into an optically scattering medium. The blind source separation technique is able to robustly identify fluorescent beads even in the presence of incomplete filtering of the scattered excitation light.

Additionally, measurement of fluorescence lifetime enables the imager to be used as an implantable sensor with the same resolution and field-of-view achieved without lifetime discrimination. In particular, we use the dependence of fluorescent lifetime on pH of fluorescein to demonstrate pH sensing capabilities. As fluorescein suffers from reduced quantum yield with decreasing pH, the development of improved pH sensitive fluorescent labels will aid in measurements of large cell populations.

The effect of tissue scattering was investigated ex-vivo through 150 μm thick whole brain slices. Here, we observe an approximate 2× reduction in resolution for the same fluorescent sources when moving from agar to neural tissue. The false count rate also increases by approximately 33%.

Ultimately, incomplete rejection of the laser excitation background photons restricts resolution, frame rate and source density. Improved spectral filters are required to image smaller, less bright fluorescent sources. We estimate that an excitation rejection of OD6 is ultimately necessary for detecting somas labeled with GFP in vivo, requiring an additional OD2 of filtering on top of the current implementation. The resulting increased SNR should allow framerates above 10 Hz. Better spectral filters will also allow for better temporal separation of more similar lifetimes, by shifting the burden of background removal from the temporal to the amplitude domain, improving sensing resolution based on fluorescent lifetime changes. Background rejection through temporal means can be further improved by reducing autofluorescence and autofluorescence lifetime in the filter dye material, improving SPAD IRF, and exploiting larger fluorophore lifetimes.

In neural tissue, scattering and absorption limit the depth of field^51^; optical power typically drops 90% at a distance of 200μm from the source. This makes light delivery at depth challenging. These rigid shanks could be co-inserted with a cannula, waveguide arrays^65^, or tapered fiber^66,67^ coupled to a mode-locked laser. Alternatively, light emitters, such as light-emitting diodes, could be cointegrated on the shank. In all cases, increasing the optical power is only of benefit if the excitation-wavelength background due to scattered photons can be sufficiently rejected. The imaging depth of field is also limited by the angular resolution of the pixels, degrading resolution at larges distances from the shank

MIMAS enables multispectral detection without requiring separate pixels for each color, as is typically done in imagers. MIMAS combines light-field imaging with pixel-to-pixel color filtering, greatly enhancing resolution over conventional angle-sensitive designs, while blocking background excitation light in the context of fluorescent imaging. MIMAS sets no requirements on the detector and adds negligible thickness, enabling many spectral sensing applications for light-field imaging, especially in applications where total imager volume is highly constrained.

## Materials and methods

### FDTD simulation

The MIMAS structure is simulated in a finite-difference time-domain (FDTD) simulator (Lumerical). The structure is idealized in four ways to enable this analysis. First, a 2D (X-Z) simulation is performed to achieve acceptable simulation time. Secondly, the use of periodic boundary conditions gives an infinitely periodic structure in the *x*-direction, eliminating edge effects. Furthermore, we assume perfect sidewalls for both the cavity oxide and phase gratings. Finally, nonidealities introduced by the CMOS detector are ignored by considering the structure on an SiO_2_ substrate. Creating a model for the CMOS stackup was found unnecessary for verification of the measurements. The source is modeled as a plane wave, as the source-to-detector distance is assumed much larger than the detector dimension. To correct for phase artifacts in FDTD-solutions, the BFAST plane wave is selected. An automatic mesh generation algorithm with Quality Level 6 is selected, with maximum mesh size of 10 nm, corresponding to ${{\Delta }}_{{{{{x}}}},{{{{z}}}}} < \frac{\lambda }{{20n}}$ for all materials. The angular, spectral, and pitch-sweep resolutions are selected to be 2°, 10 nm and 50 nm, respectively. To simulate previously proposed dielectric-phase and metal-grating angular-sensitive design^19^, the MIM analyzer grating is replaced with 200 nm thick metal, emulating grating implementations in CMOS interconnect.

### Transmission efficiency

The transmission efficiencies of devices fabricated on a silica substrate (Fig. 1b) are measured using a Newport S8 and R1936 power meter. The illumination was provided by an NKT EXU-6PP mode-locked supercontinuum laser, and wavelength selection performed by a SuperK Varia acousto-optical tunable filter (AOTF).

### Near-field Talbot effect

The nanoscale near-field Talbot measurement (Fig. 1c) was performed on a near-field scanning optical microscope (NSOM, NTMDT Ntegra system) equipped with two laser sources with wavelengths of 532 and 660 nm. An amplitude measurement was done with the NSOM in transmission mode as depicted in Fig. 5a. A cantilever with clear aperture of (70 ± 20 nm) was positioned proximate to the MIM grating structure surface. Local electric field transmits through the cantilever clear tip, and couples into a fiber through a 100x objective and its amplitude is recorded using a PMT. The cantilever, mounted on a piezo stage, scanned the local light intensity in a volume of 4 × 4 × 4 µm^35^, with lateral (*Δx*, *Δy*) resolutions of 22 nm and vertical resolution (*Δz*) of 250 nm.

The *x*-locations of peak intensities in the Talbot image were detected in each *y*-slice to align the data before subsequent averaging over all *y*, reducing measurement noise and introducing tolerance to sample rotational misalignment. The image is interpolated in the *z*-direction to match the *x*–*y* resolution. Bending of the Talbot image in the 532-nm measurement is attributed to drift in the piezoelectric elements, as a volume acquisition took 45 minutes to complete.

### MIMAS Fabrication

Sputtering of Ag (AJA Orion 8) resulted in superior film quality (pinholes, reflectivity, and adhesion to CMOS foundry passivation) when compared to e-beam-evaporated films. As a dielectric, hafnia provides a high refractive index, while being relatively easy to etch as compared to other high-κ dielectrics such as TiO_2_. The MIM grating etch mask was defined in 325-nm-thick PMMA patterned using electron-beam lithography (Elionix ELS-G100), followed by a 30 nm reactive ion etch (Oxford PlasmaLab 100 ICP) in CF_4_:Ar with a 25:25 SCCM flow ratio. The absorption filter dye (LP500, Adam Gates Company) was dissolved in cyclopentanone and mixed with SU-8 3010 negative photoresist. The SU-8 was patterned at a 3.5 µm thickness using deep UV photolithography, unaffected by the absorption filter dye.

Binary phase gratings diffract maximally when the optical path length difference is $\frac{\lambda }{4}$. The diffraction efficiency (*η*_dif_) is given by

$$\eta _{{\rm{dif}}} = \sin ^2\left[ {\frac{{2\pi d\left( {n_{{\rm{PG}}} - n_{\rm{s}}} \right)}}{\lambda }} \right]({{{\mathrm{M1}}}})$$

where *n*_S_ and *n*_PG_ are the refractive indices of the source medium and phase grating, respectively. A high refractive index contrast with the source medium (*n*_PG_*-n*_s_) is required to achieve high *η*_dif_. The phase grating is defined using mr-EBL 6000.3 resist (*n*_PG_ ≥ 1.6). Adhesion to the underlying crosslinked SU-8 was achieved by 10 minutes of oxygen plasma ashing, followed by spin coating HMDS until dry, immediately before spin-coating mr-EBL. The grating structures were patterned beyond the area of the SPAD to ensure no unmodulated light transmits to the photodetector.

Before MIMAS fabrication on the shank structures, we define the shank contour by etching 100 µm deep trenches using an IPG Cu-vapor laser. After MIMAS fabrication, we mechanically grind away excess backside silicon to a thickness of 50 µm, releasing the shanks from the surrounding die^9^. This step is performed with the Allied Tools XPREP.

### Angular modulation of MIMAS on CMOS SPAD

Measurement of relative photon transmission efficiency as a function of angle was performed as shown in Fig. 5b. A Xenon arc lamp, followed by a Spectral Products cm110 monochromator generates wideband, randomly polarized, spectrally tunable light and is coupled into a 50/50 spliced multimode fiber. The first output couples to a Thorlabs S130C+PM100D photodiode monitoring photon flux; the second output connects to a Thorlabs large beam collimating lens projected onto the MIMAS imager surface. The monochromator output bandwidth is tuned to 10 nm, achieving a measurement SNR of 40 dB while remaining well within the FWHM of the MIM resonance filter. The angular resolution of the measurement is set at 2°, while chromatic divergence of the collimator and stage uncertainty is designed to be below 0.5°.

The imager is mounted on two Thorlabs K10CR1 stages to acquire the 2D rotational modulation. The nature of the mechanics rotates the imager in spherical coordinates [ν,ζ]. The top stage holding the detector sets azimuth ζ, after which the bottom stage scans elevation ν. We prefer to display results in [θ,φ] coordinates, associated with the elevation angles pivoting around the *x* and *y* axes, which are related through the rotational transformation of ν through ζ by

$$\left[ {\begin{array}{*{20}{c}} \theta \\ \varphi \end{array}} \right] = \left[ {\begin{array}{*{20}{c}} {\cos {\upzeta}} & {\sin {\upzeta}} \\ { - \sin {\upzeta}} & {\cos {\upzeta}} \end{array}} \right]\left[ {\begin{array}{*{20}{c}} \nu \\ 0 \end{array}} \right]({{{\mathrm{M2}}}})$$

### Pinhole source measurement

To simulate fluorescent point sources, a random distribution of 8 pinholes of 25um diameter are etched into a 100 nm chrome layer on a 1 inch fused silica wafer. For both ATTO 465 and ATTO 490LS separately, we combine 30 µMol l^−1^ with 1.5% w/v agar and keep both solutions warm in the gelatinous state. Using a precision pipette, we label each pinhole with a 1 µL droplet of fluorescent agar in alternative colors. After solidifying the dispensed agar through cooling, a layer of undyed 1.5% agar is dispensed to create a uniform volume.

The NKT EXU-6PP was spectrally tuned through an NKT superK Varia to equally excite ATTO 465 and ATTO 490LS. An Olympus BX51 microscope guided the beam through the pinhole array, positioned above the imager.

### CMOS imager array and system electronics

The imager is digitally controlled by a Spartan-6 FPGA, containing a 960 MHz dynamic PLL with three-bit reprogrammable delay and fully variable duty cycle. By synchronizing onto the Supercontinuum laser 20 MHz synchronization pulse, the SPAD is time gated with 130-ps resolution in order to obtain a histogram of counts as a function of time gate delay. While the finite duty cycle of 20% (10 ns) would smear the fluorophore temporal characteristic, a convolution integral of a shifting rectangular digital shutter with an exponential fluorophore intensity decay preserves the exact exponential properties. Therefore, only shutter clock jitter (<100 ps) and FPGA system jitter (<200 ps) affect timing resolution.

The photon counts are integrated by repeating the digital shutter over 100 ms, corresponding to a frame rate of 10 Hz. Dark count rate (DCR), acquired before performing measurements is found to be 232 counts s^−1^, twice the DCR of chips prior to shank processing, but still low enough compared with the peak signal count of 50,000 counts s^−1^ in Fig. 4b. Data from hot pixels (more than five times median dark noise) are discarded. Hot pixel prevalence was found to be 3.8% for the chip used in Fig. 4.

### Brain slice extraction

Wild-type mice were anesthetized using isoflurane with an induction level of 3% and driven by an oxygen flow at the rate of 2 l/min. Upon induction of anesthesia, the mice were decapitated and the brain was extracted. 150 μm thick brain slices were acquired using the protocol described by Thomas Papouin et al.^68^. Brain slices were laminated on the imager inside a phosphate buffered saline well. Animal care and experimental protocols were approved by the Columbia University Institutional Animal Care and Use Committee.

## Supplementary information

Supplementary Information
