# Supplementary material for: Toward implantable devices for angle-sensitive, lens-less, multifluorescent, single-photon lifetime imaging in the brain using Fabry–Perot and absorptive color filters
Source: Light Sci Appl. 2022 Jan 24;11:24. doi: 10.1038/s41377-022-00708-9 (PMC8786868; doi:10.1038/s41377-022-00708-9)
Supplement: Supplementary file 1 — Supplementary Information [file 41377_2022_708_MOESM1_ESM.docx]

**Supplementary Information for** **Toward implantable devices for angle-sensitive, lens-less, multi-fluorescent, single-photon lifetime imaging in the brain using Fabry-Perot and absorptive color filters**

**Adriaan J. Taal^1^, Changhyuk Lee^2^, Jaebin Choi^1^, Björn Hellekamp^1^, and Kenneth L. Shepard^1^**

*^1^ Electrical Engineering, Columbia University, New York 10027, US*

*^2^ Brain Science Institute, Korea Institute of Science and Technology, Seoul 02792, South Korea.*

# **S1. Parameter table for FDTD simulations**

A finite difference time domain (FDTD) simulation is constructed to validate the fabricated angle sensitive front end. A screenshot of the structure in Lumerical FDTD-solutions (Fig. S1a) is shown next to the structure illustration (Fig. S1b) and the FIB-SEM cross-section after fabrication on CMOS (Fig. S1c). Table S1 outlines all simulation parameters that were held constant in all simulations, unless explicitly stated. The parameters varied in most simulations are *p*, *θ*, *Δd*, and polarization.


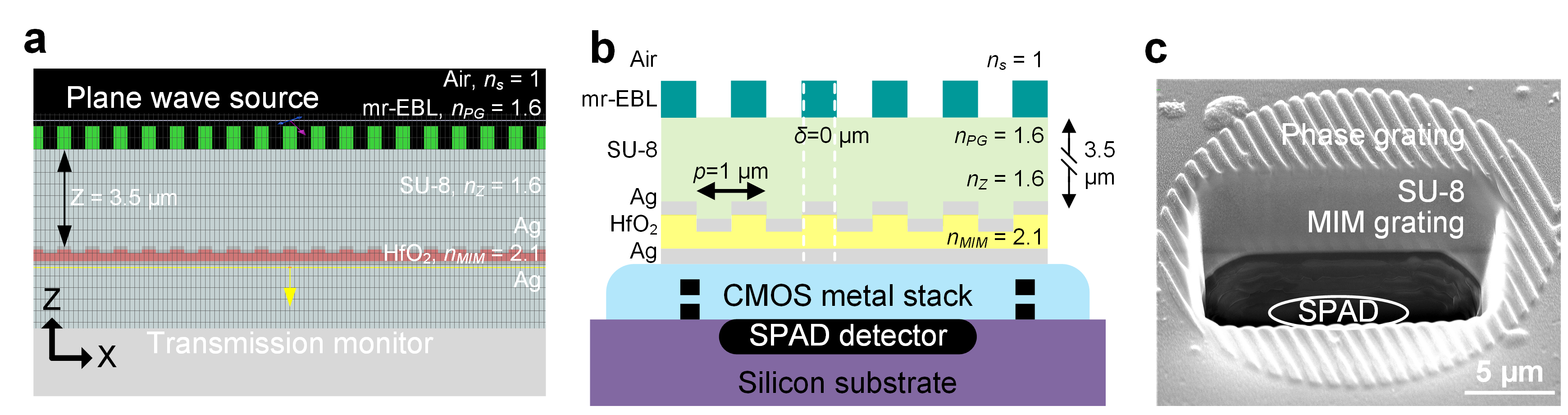


**Figure S1. a**. FDTD layout of simulation **b**. fabrication schema **c**. FIB-SEM cross-section

**Table S1.** Simulation parameters for Lumerical FDTD-solutions

| **Parameter** | **Value** | **Parameter** | **Value** |
| --- | --- | --- | --- |
| MIM metal height and material | 30 nm Ag | Simulation type | 2D, in the *x-z* plane,  Conformal variant 1 |
| MIM insulator height (*d*) and material | *d_1_*=70 nm HfO_2_, green filter  *d_2_*=100 nm HfO_2_, red filter  *n_MIM_* = 2.1 | FDTD mesh quality | Setting 6, with maximum mesh size of *λ*/(20×*n_MIM_*) nanometer |
| Separation between MIM and phase grating (*Z*) | 3500 nm SU-8, *n_Z_* = 1.6 | Polarization | Random |
| Phase grating height | 200 nm, *n_PG_*=1.6 | Monitor geometry | Line geometry along *x* |
| Simulation width (*X*) | 4*p* repetitions | Monitor recording method | Transmitted power measurement, normalized to source intensity |
| Boundary conditions | Periodic in *x* (along *z*-axis)  Perfectly matched layer (PML) in *z* (along *x*-axis) | Simulation time | 250 fs |
| Source type | Broadband fixed angle source technique (BFAST) plane wave along *x* | Source spectrum | 400 nm to 800 nm, optimized for fast pulse |
| Source medium | Air, *n_s_*=1 |  |  |

**S2. Dual bandpass filter spectral separation**

To design a dual bandpass filter frontend, we need to explore the minimum required spectral separation (Δλ) between the two bandpass filter peak wavelengths. This spectral separation is a function of Δd and n_MIM_:

$$\Delta\lambda=\lambda_{1}-\lambda_{2}=2n_{MIM}\Delta d (S1)$$

To quantify this minimum spectral separation, we look at the resulting modulation index (m) of the MIMAS structure, calculated from the minimum and maximum angular transmission (T):

$$m=\frac{\max\left( T \right)-\min\left( T \right)}{\max\left( T \right)} (S2)$$

A value for m close to 1 indicates high sensitivity to shifts in incident angle, increasing spatial resolution. Fig. S2 shows a simulation of Δλ versus m. One cavity thickness is held constant at 95 nm to create a transmission peak at 600 nm, while Δd is swept to vary the second bandpass filter wavelength between 400 and 800 nm.

Very high modulation indices (*m*>0.8) can be achieved when both wavelengths of peak transmission are separated by more than 75 nm. When *Δλ* is equal to 60 nm, i.e. equal to the FWHM of the color bandpass filter (Fig. 1B), the resulting modulation index is decreased to exactly one half. Below this limit, the MIM grating effectively transforms into a planar MIM and stops generating a Talbot diffraction pattern.


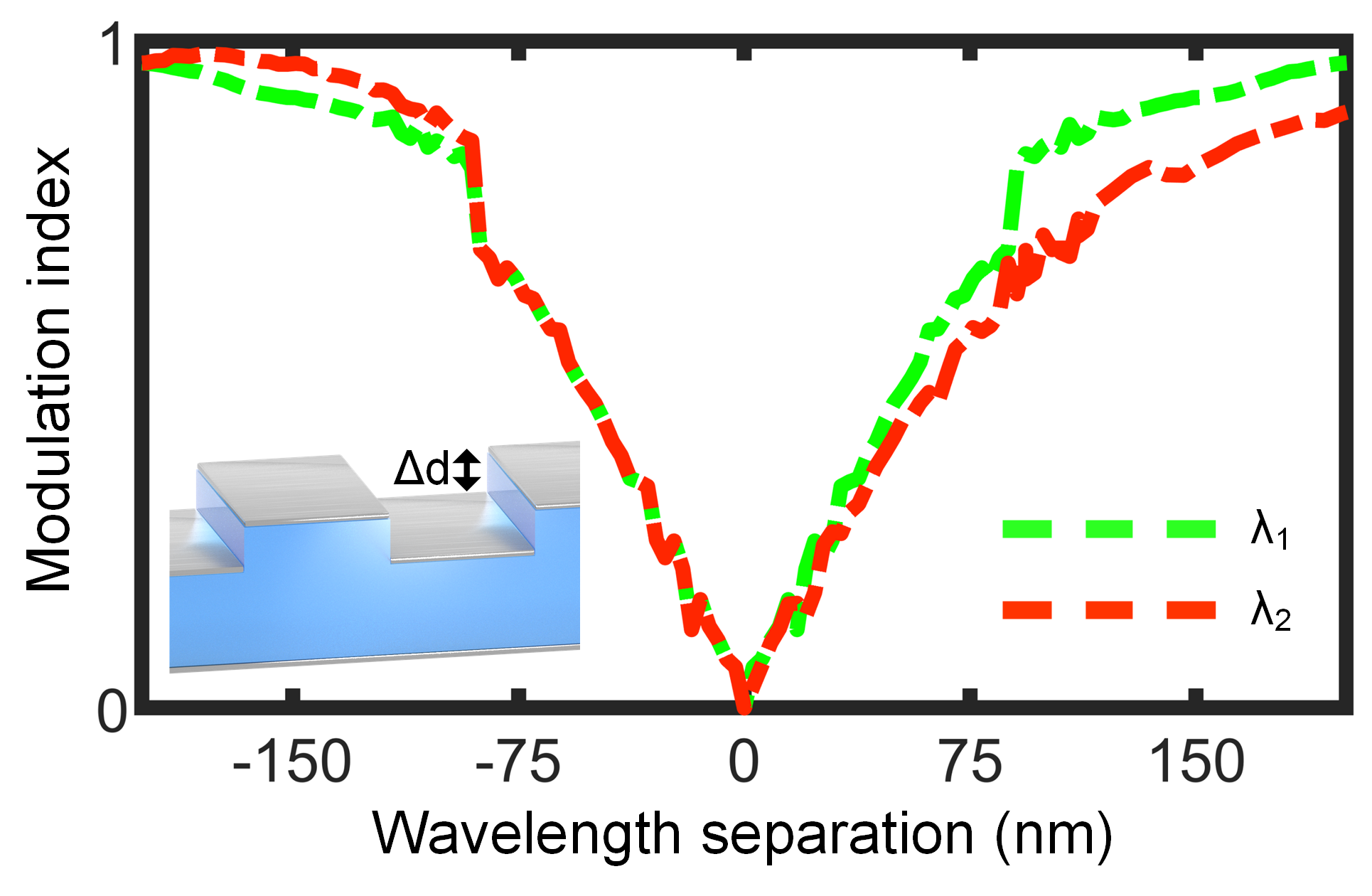


**Figure S2.** FDTD simulation showing modulation index (m) of MIMAS frontend versus wavelength separation (*Δλ* = *λ_1_* - *λ_2_*) directly resulting from etch depth *Δd*. The red wavelength of maximum transmission *λ_2_* is held constant at 600 nm, and the MIM insulator thickness difference *Δd* is varied.

# **S3. Robustness engineering of MIM transmission spectrum**

For a Fabry-Perot transmission filter, the wavelength of maximum transmission *λ_0_* is shifted by changes in both the grating pitch and incident angle. The wavelength of peak transmission as a function of incident angle depends on *n_s_* and *n_MIM_*:

$$\lambda_{0}\left( \theta\right)=\lambda_{0}\left( \theta=0 \right)\sqrt{1-\frac{n_{s}^{2}\sin^{2} \left( \theta\right)}{n_{\mathrm{MIM}}^{2}}} \text{(S3)}$$

Additionally, the incident angle increases the filter spectral FWHM:

$$FWHM\left( \theta\right)=\frac{FWHM\left( \theta=0 \right)}{\sqrt{1-\frac{n_{s}^{2}\sin^{2} \left( \theta\right)}{n_{\mathrm{MIM}}^{2}}}} \text{(S}\text{4}\text{)}$$

An increasing incident angle therefore decreases the Q-factor (Eq. 1). This effect is countered by selecting HfO_2_ as the insulator material with its high refractive index (*n_MIM_*=2.1, Fig. S3a). The measured peak transmission wavelength and FWHM shift less than 3% for incident angles up to 30° (Fig. S3b).

The dual-bandpass characteristic (centered at two wavelengths, *λ_1_* and *λ_2_*, with *λ_2_* > *λ_1_*) of the MIMAS filter is also impacted by lateral cavity dimensions, equal to half the pitch.

We simulate the spectral transmission efficiency for different grating pitch (Fig. S3c). The observed shift in peak transmission wavelength is less than 10 nanometers for either wavelength (*λ_1_*, *λ_2_*).

As the optical lengths are determined by the refractive index, using the high-index insulator (HfO_2_) allows physical dimensions to be scaled. By permitting a smaller pitch, the range of available angular frequencies (*β*) in a MIMAS device is increased.


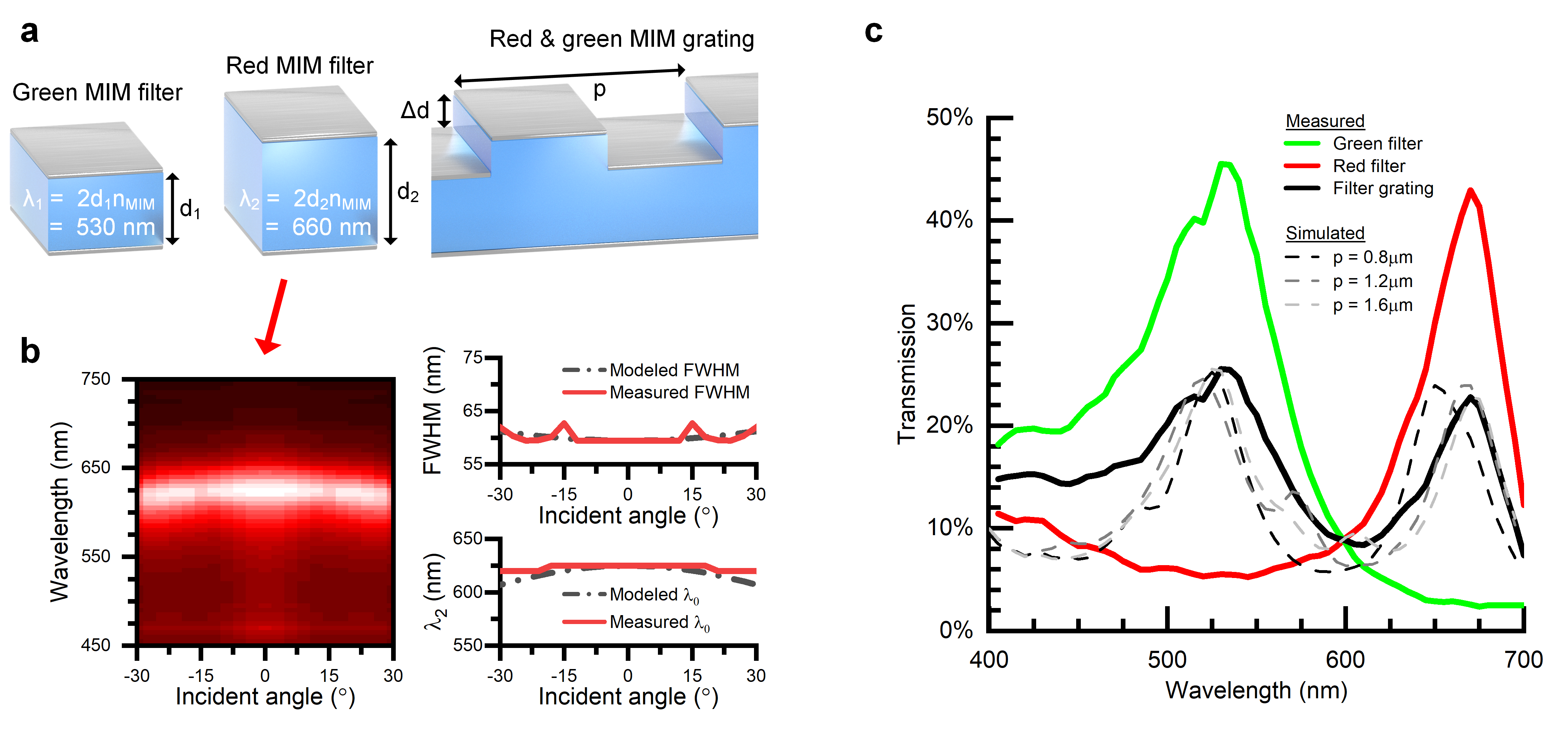


**Figure S3.** **a.** Sketch of MIM grating structure consisting of interleaved Ag-HfO2-Ag color filters with measured individual resonant frequencies of 530 and 660 nm. **b.** Stability of transmission characteristics under incident angle for red bandpass filter. **c.** Transmission efficiencies of individual bandpass filters and interleaved devices over the visible spectrum for measured (solid) and FDTD simulated (dash) devices. The transmission spectrum of the grating filter device is agnostic to pitch.

# **S4. Grating transmission NSOM measurement versus FDTD simulation**

Fig. S4a illustrates how near-field scanning optical microscopy (NSOM) is performed in free space. Fig. S4b shows the NSOM measurement result in an area of 4 by 4 μm above the MIM grating, which are compared with simulation in Fig. S4c. For the grating with 1µm pitch in air (*n_Z_*=1), the first Talbot order (*i*=1) is found at a depth of 1.9 μm and 1.5 μm for wavelengths of 532 nm and 660 nm respectively. The second order (*i*=2) is located at 3.7 μm and 3 μm. To validate measurement and simulation, we compare results with the predicted Talbot distance:

$$Z_{T,i}\left( \lambda,n_{Z},p \right)= i\cdot Z_{T,i=1}=i\cdot\frac{{n_{Z}p}^{2}}{\lambda} (S5)$$

which yields Talbot distances of 1.9 μm and 1.5 μm for the wavelengths of 532 nm and 660 nm, respectively, which agree with measured and simulated values.


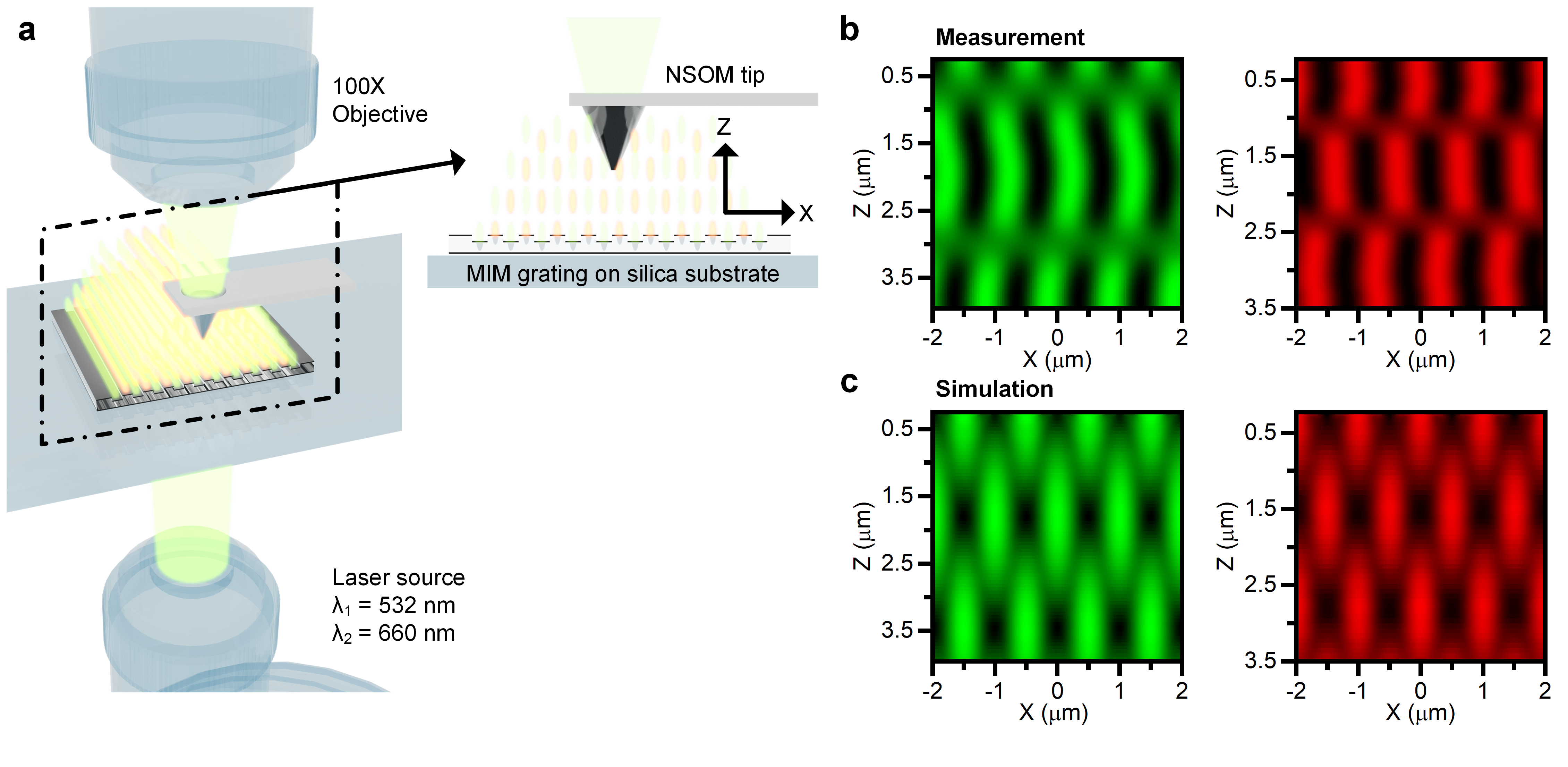


**Figure S4.** Generation of Talbot self-image generated by the device at green and red wavelengths verified using Near Field Scanning Optical Microscopy (NSOM). **a.** The nanoscale near field Talbot measurement was done on a NTMDT Ntegra equipped with two laser sources with wavelengths of 532 and 660 nm. An amplitude measurement was done with the NSOM in transmission mode as depicted. **b.** NSOM measurement for wavelengths of 532 nm and 660 nm. **c.** FDTD simulation showing agreement with measured device.

**S5. Analytical modeling of 2D angular modulation function**

Defining a correct model for the MIMAS spatial compression is important to achieve correct image inversion. We construct the compression matrix *A* from the angular modulation as follows. The imaging volume of interest is first defined in an arbitrary voxel grid. We rotate the voxel grid, relative to the pixel location (*x_0_, y_0_*), according to the grating orientation χ. Each voxel in the imaging volume is then mapped from cartesian coordinates [*x*,*y*,*z*] to elevation in the directions perpendicular (*θ*) and parallel (*φ*) to the gratings:

$$\left[ \begin{matrix} \theta\\ \varphi\end{matrix} \right]=atan2\left( z, \left[ \begin{matrix} \cos\chi& \sin\chi\\ -\sin\chi& \cos\chi\end{matrix} \right]\left[ \begin{matrix} x-x_{0} \\ y-y_{0} \end{matrix} \right] \right)-\frac{\pi}{2} (S6)$$

The pixel spatial response is calculated for a given wavelength *λ* as:

$$T\left( \nu,r,\theta,\lambda\right)=\frac{F\left( \nu\right)}{r^{2}}\cdot\left( \frac{1}{2}+\frac{m}{2}\cos\left[ \beta\left( \lambda\right)\cdot\theta+\alpha\left( \lambda\right) \right] \right) (S7)$$

where *ν* and *r* are the elevation and radius from converting [*x-x_0_*, *y-y_0_*, *z*] to spherical coordinates. The angular frequency *β* and angular offset α are wavelength-dependent (eq. S15) for the MIMAS frontend. The windowing function $F$ is determined by the angular response of the SPAD detector, independent of the gratings:

$$F\left( \nu\right)=\sin^{2} \left( \gamma\nu\right) (S9)$$

We found *γ*=3/2 works well for all fabricated geometries, reducing photon sensitivity to zero for $\left| \theta\right|>60^{\circ}$, similar to previously characterized angle sensitive structures^1^. This maximum angle of incidence is determined by the numerical aperture of the SPAD detector.

Sixteen geometries are repeated 64 times across the 1024 pixels. Figure S5 plots the angular modulation for green (*λ*=532nm) and red (*λ*=660nm) wavelengths for the two geometries displayed in Figure 2d. The pixel-to-pixel fabrication standard deviation in *m* is typically less than 0.06.


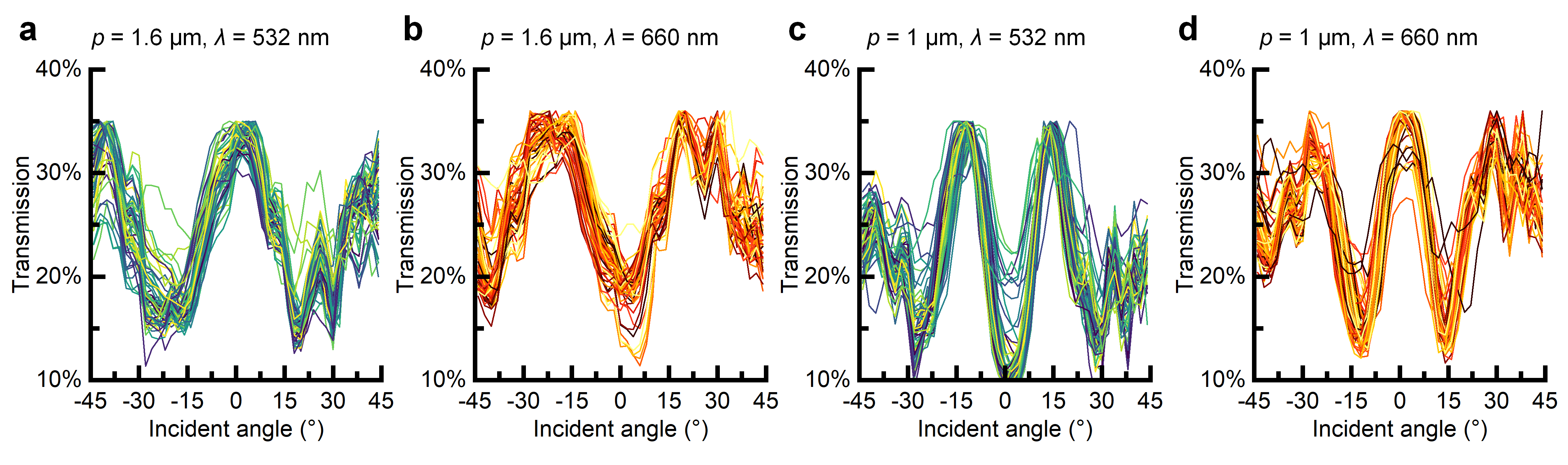


**Figure S5.** Angular responses of all 64 pixels of the same geometry for **a.** low angular frequency, λ=532nm **b.** low angular frequency, λ=660nm **c.** high angular frequency, λ=532nm **d.** high angular frequency, λ=660nm

# **S6. Effect of front-end geometries on source separability**

The linear source separability of the imager is determined by chosen combinations of MIMAS front-end geometries. Finding a layout which maximizes the separability is computationally infeasible. Instead, we search for a suitable layout by elimination of parameters. To simplify the design space considerably, we optimize for a combination of 16 MIMAS geometries. The combination is then repeated over the 1024-pixel imager. Fluorescent sources, at distances of 100 – 200 µm away from the imager, will be in the field of view of all different 16 MIMAS geometries.

In Fig. S6a, we start with a complex layout; the 16-pixel ensemble is created from 16 grating orientations (*χ*), four angular frequencies (*β*), two complementary grating offsets (*α*). At this pixel pitch, grating offsets (*α*) do not contribute to separability, as shown in Fig. S6b by elimination. A similar argument is found in Fig. S6c for reducing the number of angular frequencies (*β*) to two. Implementing high values for angular frequencies (*β* > 12, Fig. S6c) leads to better separability than lower angular frequencies (*β* < 12, Fig. S6d). Rotations are crucial for linear separability (Fig. S6e). Finally, the comparison is made to previous angular sensitive design^2^ (Fig. S6f).


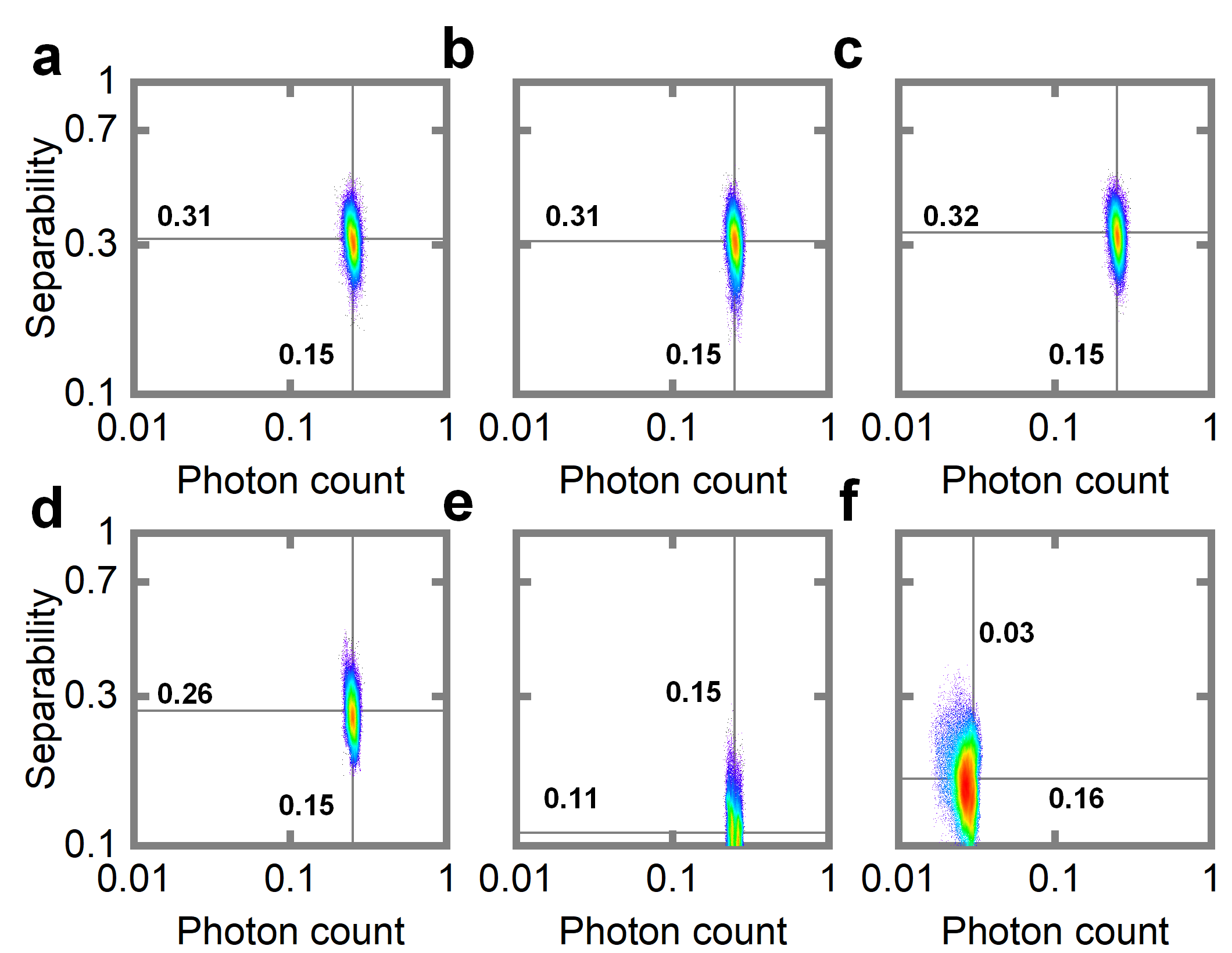


**Figure S6.** Linear separability of the layout; **a.** with 16 grating orientations, four angular frequencies, two grating offsets, **b**. without grating offsets, **c**. without offsets, with two high values for angular frequency, **d**. with two low angular frequencies, **e**. without rotations. **f**. Comparison with previous angle sensitive design.

# **S7. Spectral sensitivity of angle-sensitive designs**

In Figs. 3, 4, and 5, we demonstrated how angular modulation enables 3D localization of point sources by solving a least squares linear system. The submatrices of this system for each color ($A_{g}$, $A_{r}$) are constructed by mapping each voxel to each pixel’s spatial response function. The degree of orthogonality between the two submatrices quantifies the capability for spectral identification. This allows for system-wise comparison of multispectral reconstruction between a conventional angle sensitive design and the MIMAS structure.

The FDTD simulation of the angular transmission spectrum, *T(θ,λ)*, for a metal-based angle-sensitive (AS) design (Fig. S7a) shows little change in modulation from green (*λ_1_*=530nm) to red (*λ_2_*=660nm). The submatrices $A_{g}$ and $A_{r}$ will thus be highly correlated. Instead, for MIMAS, the out of phase angular transmission spectrum (Fig. S7b) forces orthogonality between its submatrices.

We quantify orthogonality between the submatrices by calculating their inner product:

$$\left\| A_{g}^{MIMAS}\cdot A_{r}^{MIMAS} \right\|_{2}^{2}<\left\| A_{g}^{AS}\cdot A_{r}^{AS} \right\|_{2}^{2} \text{(S10)}$$

yielding normalized values of 0.085 for MIMAS, and 0.17 for the conventional angle-sensitive front end for the pinhole imaging problem (Fig. 5). The red MIMAS submatrix is 47% more orthogonal to its green counterpart, significantly improving spectral identification compared to metal-based angle-sensitive design.


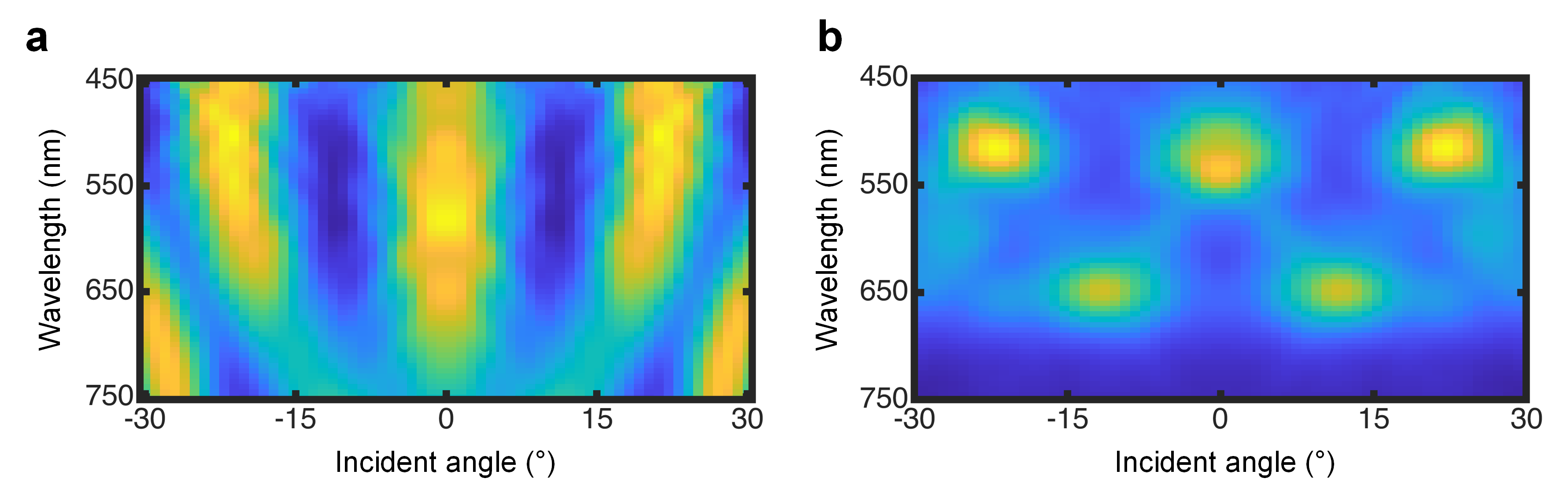


**Figure S7.** FDTD simulations of the angular transmission spectrum, for (**a)** conventional AS front end using a metal and phase grating, (**b)** MIMAS front end.

# **S8. Robustness of source localization result**

To demonstrate the robustness of the localization solution presented in Fig. 5e, we demonstrate an unchanging solution when increasing the cardinality *L*. We solve for the exact locations by fitting the image to our recorded data (Fig. S8a) using the 1-norm constrained least squares solver. To arrive at the desired solution cardinality *L*, adjust the sparsity parameter *λ*. After the exact locations are found, an inverse image is formed by multiplying the solution vector $\hat{x}$ with the projection matrix *P_A_*.

The optimal solution is found for *L*=8 (Fig S8b), when all locations are uniquely found. By increasing *L* to 12 (Fig. S8c) we find a similar image, with the four additional locations closely neighboring the original locations. Only when L is increased to 16 (Fig. S8d), we find one location at [*x*,*y*] = [1700, 800] µm where the red and green channel overlap.


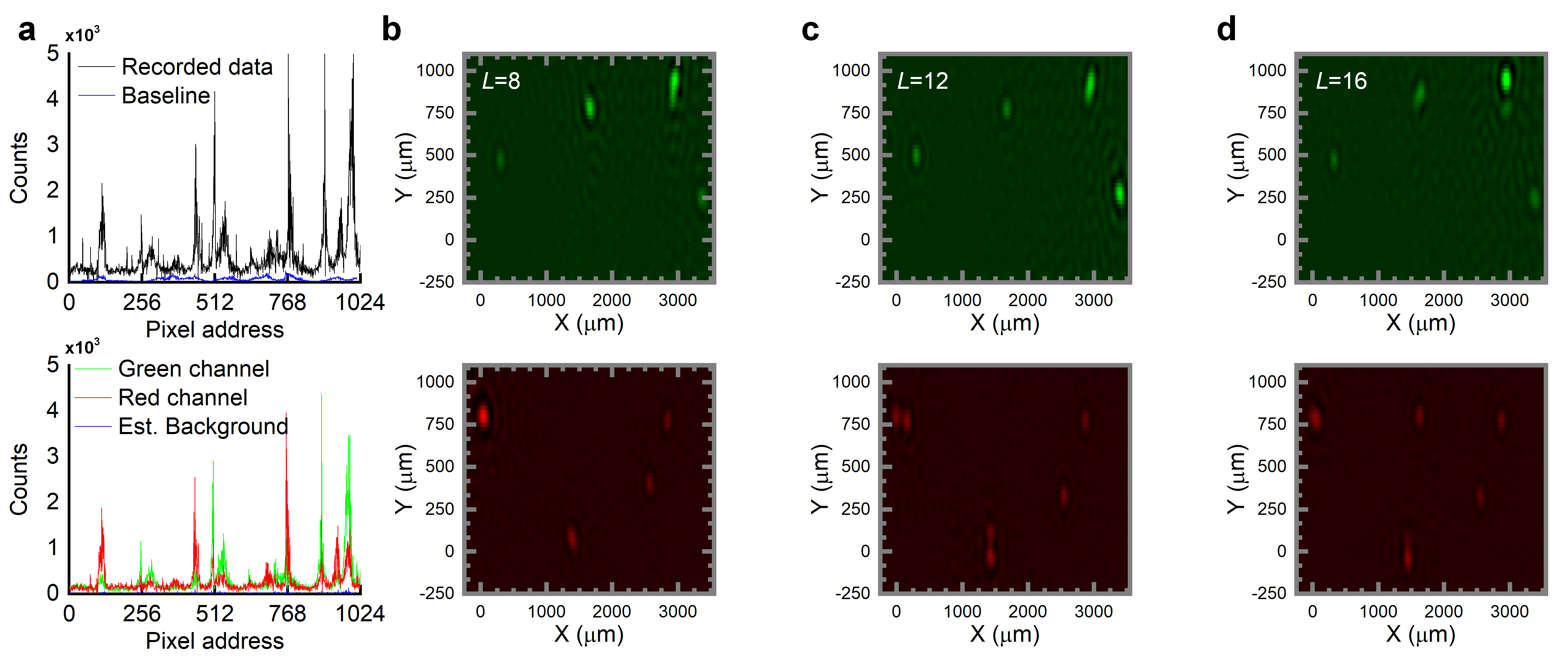


**Figure S8.** Robustness of image reconstruction (**a)** Recorded data from the fluorescent pinhole experiment, compared to a baseline in the absence of fluorescent dye. The data is separated into green and red color channels after blind source separation. Reconstruction results versus desired sparsity (*L*) for (**b)** *L*=8 **(c)** *L*=12 **(d)** *L*=16.

# **S9. Point spread function sensitivity to system noise and wavelength deviation**

Both source spectral uncertainty and electronic noise affect resolution in the image reconstruction. While this analysis was previously performed for electronic noise^3^, we extend the analysis to include the contribution of spectral uncertainty on angular estimation.

We consider a single point source sampled by four angle sensitive pixels. These pixels have equal modulation frequency (β); each phase (α) is offset by 90°. In order to determine the incident angle (*θ*), the relative photon count from each of the four phases (y_α_) can be combined into a quadrature signal (*I_quad_*) of the form:

$$I_{quad}=\frac{y_{\alpha=270^{\circ}}-y_{\alpha=90^{\circ}}}{y_{\alpha=0^{\circ}}-y_{\alpha=180^{\circ}}}=\left[ \frac{m\cos\left( \beta\theta+270^{\circ} \right)-m\cos\left( \beta\theta+90^{\circ} \right)}{m\cos\left( \beta\theta\right)-m\cos\left( \beta\theta+180^{\circ} \right)} \right]+\epsilon=\tan\left( \beta\theta\right)+\epsilon(\text{S}12)$$

Noise, either Poisson or additive Gaussian, is represented by $\epsilon$ with variance $\sigma_{\epsilon}^{2}$. *I_quad_* has an SNR of $\frac{2m^{2}}{\sigma_{\epsilon}^{2}}$.

First, in order to derive the effect of wavelength deviation, we set noise ($\epsilon$) to zero. We analyze the effect of a source spectral variance ($\sigma_{\lambda}^{2}$) on the angular estimation error ($\sigma_{\theta}^{2}$) and, subsequently, the spatial estimation error ($\sigma_{x}^{2}$). For balanced quadrature sensing,^3^ the estimated angle $\hat{\theta}$ is the arctangent of *I_quad_*, divided by the *a priori* characterized angular frequency ($\beta_{d}$):

$$\hat{\theta}=\frac{1}{\beta_{d}}\mathrm{atan} \left( \frac{y_{\alpha=270^{\circ}}-y_{\alpha=90^{\circ}}}{y_{\alpha=0^{\circ}}-y_{\alpha=180^{\circ}}} \right)=\frac{1}{\beta_{d}}\mathrm{atan} \tan\beta\theta=\frac{\beta}{\beta_{d}}\theta\text{(S13)}$$

When the *a priori* assumed wavelength is exactly equal to the source wavelength *λ*, the characterized angular frequency $\beta_{d}$ matches the true angular frequency $\beta$:

$$\beta\left( \lambda\right)=\frac{2\pi n_{s}}{pn_{Z}}\cdot i\cdot\frac{{n_{Z}p}^{2}}{\lambda} \left( S14 \right)$$

In any realistic experimental system, however, the assumption of sources with infinitely narrow spectra does not hold. A stochastic spectral spread is inherent to both fluorescent dyes and quantum dots.^4^

With varying wavelength ($\partial\lambda$), the true angular frequency $\beta$ varies, inducing a misestimate in angle. The angular sensitivity with respect to wavelength $\left( \frac{\partial\theta}{\partial\lambda} \right)$ depends on *θ* and *λ* and, therefore, angular frequency:

$$\frac{\partial\hat{\theta}}{\partial\lambda}=\frac{\partial\beta}{\partial\lambda}\cdot\frac{\theta}{\beta_{d}}=-\frac{\theta}{\lambda} \left( S15 \right)$$

The angular error relative to the angle of incidence $\left( \frac{\partial\hat{\theta}}{\theta} \right)$ is negatively proportional to the relative wavelength shift $\left( \frac{\partial\lambda}{\lambda} \right)$. By modeling the source spectrum as a normal distribution with variance $\sigma_{\lambda}^{2}$, the angular estimation is normally distributed with unbiased mean ($\mu_{\theta}$) and scaled variance ($\sigma_{\theta}^{2}$):

$$\hat{\theta} \sim N\left( \mu_{\theta}=\theta,\sigma_{\theta}^{2}\left( \lambda\right)=\frac{\theta^{2}}{\lambda^{2}}\sigma_{\lambda}^{2} \right) (\text{S16)}$$

To quantify the angular estimation error caused by electronic noise ($\sigma_{\epsilon}^{2}$,) we take the derivative^3^ of Eqn. S13:

$$\sigma_{\theta}^{2}(\epsilon)=\left| \frac{d\theta}{dI_{quad}} \right|^{2}\times\sigma_{\epsilon}^{2}=\left( \frac{\cos^{2} \left( \beta\theta\right)}{\beta} \right)^{2}\times\frac{2m^{2}}{SNR} (\text{S}17)$$

where $\beta$ depends on wavelength. For simplicity we assume the contributions to angular error from spectral uncertainty (Eqn. S16) and electronic noise (Eqn. S17) are uncorrelated. The total angular estimation variance is the sum of both variances:

$$\sigma_{\theta}^{2}=\sigma_{\theta}^{2}\left( \epsilon\right)+\sigma_{\theta}^{2}(\lambda)=\left[ \frac{\theta^{2}}{\lambda^{2}}\sigma_{\lambda}^{2}+\left( \frac{\cos^{2} \left( \beta\theta\right)}{\beta} \right)^{2}\times\frac{2m^{2}}{SNR} \right] (\text{S}18)$$

The spatial error ($x_{e})$ at any vertical distance (*z*) is calculated from the tangent of the estimated angle, influenced by the sampled noise and wavelength deviation:

$$x_{e}=x-\hat{x}=z\tan\theta-z\tan\hat{\theta}=z\tan\theta-z\tan\left( \theta-\frac{\theta\partial\lambda}{\lambda}-\frac{\cos^{2} \left( \beta\theta\right)}{\beta}\times\epsilon\right) (\text{S19)}$$

The spatial point spread function ($\sigma_{x_{e}}^{2}$) then resolves to a hyperbolic tangent:

$$\sigma_{x_{e}}^{2}=\text{var}\left[ z\tan\left( \hat{\theta} \right) \right]=z^{2}E\left\{ \frac{1-\cos2\hat{\theta}}{1+\cos2\hat{\theta}} \right\}=z^{2}\frac{1-e^{-\sigma_{\theta}^{2}}}{1+e^{-\sigma_{\theta}^{2}}}=z^{2}\tanh\sigma_{\theta}^{2}=z^{2}\tanh\left( \frac{\theta^{2}}{\lambda^{2}}\sigma_{\lambda}^{2}+\left( \frac{\cos^{2} \left( \beta\theta\right)}{\beta} \right)^{2}\times\frac{2m^{2}}{SNR} \right) (\text{S20)}$$

A higher angular frequency (β) results in higher resolution (smaller $\sigma_{x_{e}}^{2}$), at the cost of higher sensitivity to spectral deviation. Additionally, the quadrature angular estimation is more sensitive to spectral deviation at higher incident angles *θ*, while the estimation is more sensitive to electronic noise at lower incident angles.

Fig. S9a plots the spatial error versus spectral FWHM and SNR. Parameters used here are *β*=20, *θ*=180°/β, *z*=150µm, *λ*=520nm, *m*=1. Detailed plots for measurement parameters (SNR = 6, filter FWHM = 60 nm) are shown in Fig. S9b. We observe that for low SNR cases, a tighter filter FWHM should be ensured to maintain resolution, and vice versa.

The 60-nm-filter FWHM (Fig. S3a) provides the benefit of increased accuracy, at the cost of signal attenuation at the extremities of the source spectrum. This is especially useful for sources with very broadband emission spectra, such as fluorescein YG (150 nm FWHM). Without spectral bandpass filtering, reconstructing such a source would incur a strongly deteriorated spatial resolution.


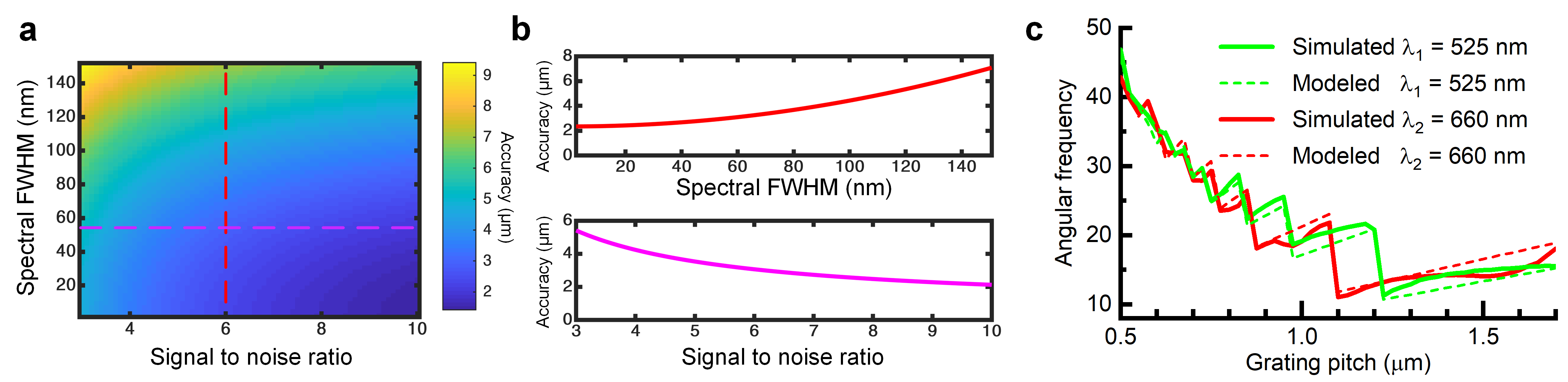


**Figure S9**. **a.** Localization accuracy versus source spectral deviation and detector Signal-to-Noise-Ratio (SNR). **b.** Detailed plots for accuracy as a function of either SNR and source spectral FWHM, for measured parameters (SNR = 6, filter spectral FWHM = 60nm). **(c)** FDTD simulation of angular frequency versus grating pitch. The modeled values are derived from equation S14.

For the 3.5 μm thick SU-8 separation medium, we can achieve a maximum angular frequency of *β*=45 (Fig. S9c). At a distance of 150μm from the imager, this will correspond to a maximum theoretical spatial resolution of Δx = 150μm × tan(180°/45) = 10 μm. The smallest possible pitch is equal to the largest wavelength within the separation material (*p* > *λ*/*n_Z_*).

**S10. Effect of scattering medium on point spread function**

We investigate the effect of strongly scattering medium on the point spread function (PSF) quality. We record the data from a single 45 μm bead located 150μm above the imager inside scattering tissue (Fig. S10a). The data is then reconstructed into a PSF using the pseudoinverse (Fig. S10b). The FWHM increases to approximately 127 μm, compared to 52 μm in the ideal case. The increase is attributed to scattering, causing pixels in a wider area to register photons from the fluorescent source.


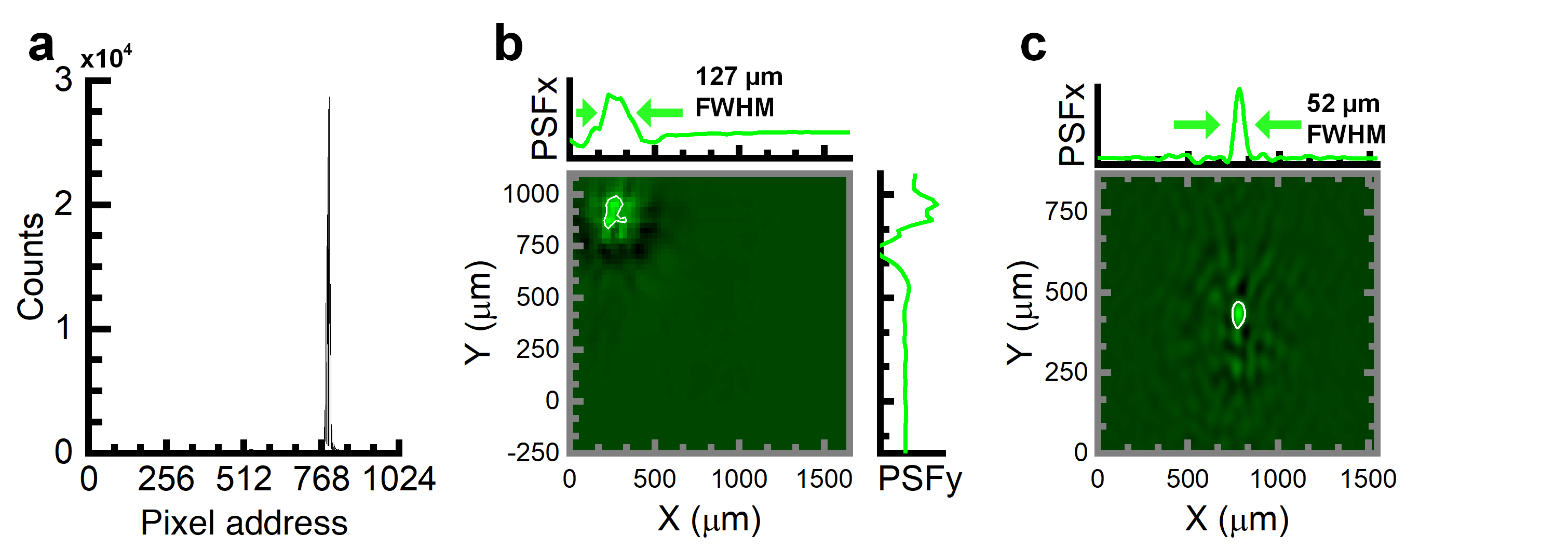


**Figure S10.** Effect of scattering medium of (**a)** Photon counts for a single 45μm bead located 150 μm above the imager inside scattering tissue (**b)** Reconstructed image for the photon counts in **(c)** Ideal PSF 150 μm above the imager.

**Supplementary References**

1. Lee, C. *et al.* 11.5 A 512-Pixel 3kHz-Frame-Rate Dual-Shank Lensless Filterless Single-Photon-Avalanche-Diode CMOS Neural Imaging Probe. in *Digest of Technical Papers - IEEE International Solid-State Circuits Conference* vols 2019-Febru 198–200 (2019).

2. Choi, J. *et al.* A 512-Pixel, 51-kHz-Frame-Rate, Dual-Shank, Lens-Less, Filter-Less Single-Photon Avalanche Diode CMOS Neural Imaging Probe. *IEEE J. Solid-State Circuits* (2019) doi:10.1109/JSSC.2019.2941529.

3. Lee, C., Johnson, B. & Molnar, A. Angle sensitive single photon avalanche diode. *Appl. Phys. Lett.* **106**, (2015).

4. Han, M., Gao, X., Su, J. Z. & Nie, S. Quantum-dot-tagged microbeads for multiplexed optical coding of biomolecules. *Nat. Biotechnol.* **19**, 631–635 (2001).
